# Optimization of the Entropy-Based Wavelet Method for Removing Strong RF and AC Interferences in a Charge Detection Linear Ion Trap Mass Spectrometer

**DOI:** 10.1021/acs.analchem.4c06069

**Published:** 2025-02-26

**Authors:** Minh Cong Dang, Avinash A. Patil, Thị Khánh Ly Lại, Szu-Wei Chou, Trang Kieu Thi Hoang, Mhar Ian Cua Estayan, Wen-Ping Peng

**Affiliations:** † Department of Physics, National Dong Hwa University, Shoufeng, Hualien 97401, Taiwan; ‡ Department of Nuclear Physics, University of Science, Vietnam National University − Ho Chi Minh City, Ho Chi Minh City 700000, Vietnam; § Department of Tracer Technique, Centre for Applications of Nuclear Technique in Industry, Vietnam Atomic Energy Institute, Lam Dong 670000, Vietnam; ∥ Department of Mathematics and Physics, University of Santo Tomas, Manila 1008, Philippines

## Abstract

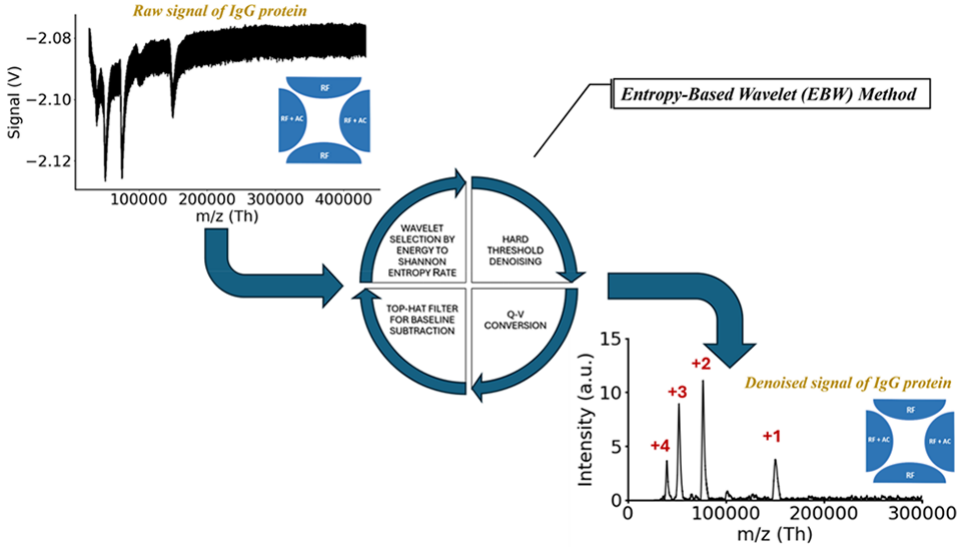

We developed an entropy-based wavelet method to effectively remove interference from strong radio frequency (RF) and auxiliary alternating current (AC) fields in a linear ion trap (LIT) mass spectrometer coupled to a charge sensing particle detector (CSPD). By optimizing the energy-to-Shannon entropy, we identified the optimal mother wavelet family and decomposition level and determined suitable threshold values based on the median of sub-band coefficients at each decomposition level. These thresholds were applied as rigid criteria across all decomposition levels to eliminate noise interferences and avoid the arbitrary choice of the threshold. This entropy wavelet-based method successfully denoised high-mass protein mass spectra, achieving significant improvements in signal-to-noise ratio (S/N) for immunoglobulin G (IgG) and alpha-2-macroglobulin (A2M) ions, with increases of 68.03% and 81.73%, respectively. Our method surpasses previously reported baseline correction techniques, such as orthogonal wavelet packet decomposition (OWPD) filtering, and enhances the sensitivity of LIT mass spectrometry (LIT-MS) in analyzing high-mass protein ions.

## Introduction

Signal processing is critical in ion trap mass spectrometry, particularly for image charge detectors operated under strong radio frequency (RF) sources, which can significantly influence the observed ion signal. Conventional smoothing and averaging techniques were widely adopted to reduce noise interference in mass spectra.^1^ Neelakantan et al.^2^ introduced the box-car average noise reduction method; however, this method easily distorts and severely deforms the signal when the window size is large. Cooley and Tukey^3^ introduced the Fourier transform (FT) and the discrete Fourier transform to perform noise filtering of signals. However, the effectiveness of noise filtering using FT is limited to signals with well-defined periodicity and stable frequencies. FT-based filtering is inefficient for processing signals containing features with varying shapes and widths (unstationary signals).^4^ To address this limitation, wavelet-based methods were developed for signal processing, providing an alternative approach for handling signals with varying frequencies.^5^ Numerous researchers have emphasized the importance of using wavelet-based methods to denoise signals.^6−11^ For instance, wavelet-based methods commonly involve selecting an appropriate wavelet from wavelet families based on “vanishing moments”. This is a widely used approach for noise reduction, where noise is removed by applying a suitable threshold. Li et al.^11^ later demonstrated that noises in the mass spectrometry data can be significantly reduced with an appropriate selection of wavelet functions and decomposition levels. Besides, Lijun et al.^6,7^ developed a wavelet-based approach to remove sinusoidal interference by applying a level-dependent threshold to the detail coefficients of the signal and selecting wavelets based on the “vanishing moment” with Daubechies wavelet families;^12−14^ however, their method is limited when mass spectra contain many ion peaks. Moreover, the wavelet-based method made it difficult for the piecewise smooth approach to simulate the shape of the original signals exactly.

Furthermore, Johnstone et al.^15^ proposed a “hard/soft threshold” wavelet method for estimating the threshold values used to separate signal from noise during the denoising process. Their findings conclude that the hard threshold method not only retains more signal features but may also introduce artifacts. In contrast, the soft threshold method provides smoother results by reducing artifacts but might slightly oversmooth the signal. Later, Chou et al.^16^ and Patil et al.^17^ introduced the orthogonal wavelet packet denoising (OWPD) method to denoise RF interference picked up by the charge detector (CD) from quadrupole ion trap mass spectrometry (QIT-MS) and rectilinear ion trap mass spectrometry (RIT-MS).^17−21^ The CD is a circular/rectangular metal disk located at the center of the detector board and utilized as a Faraday disk to obtain the image charge induced by the ions but without an interference shielding unit (Faraday cage). The diameter of the Faraday disk should be sufficient to encompass all particles ejected from the ion trap. In the prior study, the db4 wavelet from the Daubechies family, with four vanishing moments, was identified as suitable for decomposing the raw signal into detailed sublevels: cD*n* (*n* = 3, 2, and 1), and the highest approximation level cA3, and then the values of the detail sub-bands were subtracted from the corresponding estimated baseline for each level, effectively reducing the baseline to zero. This approach resulted in approximately 70% noise reduction in the signal-to-noise ratio (S/N). However, the RF interference in QIT-MS was relatively weak due to the grounding of both end-caps and the placement of a grounded plate between the end-cap and CD. Compared to QIT-MS, the noise interference was significantly stronger in RIT-MS as the CD is positioned above the RF electrode. This necessitated the development of a more advanced orthogonal wavelet packet decomposition (OWPD) denoising program to address the noise. Additionally, RIT-MS employs a voltage-scan method, where RF frequency remains fixed and only the amplitude varies during mass scanning; a wavelet-based approach that optimizes wavelet family selection and applies zero-baseline correction was still effective for denoising the mass spectra obtained by the CD.

In the present study, RF/alternating current (AC) interference in the linear ion trap (LIT) system becomes more complex when the frequency-scan method with resonance ejection is employed to scan high-mass analytes.^22^ LIT mass spectrometry (LIT-MS) instruments in frequency scan mode sweep the RF from hundreds to tens of kHz (∼300 V_p–p_) with an added AC field (∼30 V_p–p_) during resonance ejection, presenting noise analysis, information loss, and wavelet selection challenges. Traditional wavelet selection methods for reducing strong RF and AC interference have lacked evaluation in optimal decomposition levels and “hard threshold” settings. To address this, we developed a wavelet-based approach to reduce RF/AC interference in LIT-MS, focusing on selecting a mother wavelet based on energy and Shannon entropy. Median and energy values of each detail coefficient identify noise in raw ion signals, followed by thresholding at each decomposition level to improve the S/N ratios. The top-hat filter, as Stanford et al.^23^ introduced, estimates and subtracts the ion signal baseline. Analyzing immunoglobulin G (IgG) and alpha-2-macroglobulin (A2M) ions under strong RF/AC interference, our method showed an average S/N improvement of 68.03% and 81.73%, validating its efficacy in noise reduction without signal distortion.

## Experimental Section

### Instrumentation

Figure S1 (Section I in the Supporting Information) illustrates the MALDI LIT-MS instrument’s design. A commercial three-segment LIT^24^ (Thermo-Fisher Scientific, Waltham, MA, USA) was used as a mass analyzer, and the AcroMass charge-sensing particle detector^25^ (CSPD, Taipei, Taiwan) was used for ion detection as shown in Section I and Figure S1c in the Supporting Information. To prevent CSPD saturation under strong RF/AC interferences, the CSPD is shielded by an interference shielding unit (Faraday cage). The image charge of ions approaching CSPD is recorded and converted to voltage output by the charge-to-voltage conversion circuit (QVC) (Figure S2). The RF voltage was amplified by broadband high voltage amplifiers (BHVAs) (AcroMass Inc., Taipei, Taiwan), with connections depicted in Figure S1b. Mass spectra were acquired using an FPGA-based custom setup by AcroMass Inc., generating synchronized RF, DC offset, and auxiliary AC signals. An RF signal from a function generator (AFG 2005; GW Instek, New Taipei, Taiwan) was amplified and applied to the ion-guiding quadrupole Q1 (P/N: 97055e20106, by Thermo-Fisher Scientific, Waltham, MA, USA) and monitored by Agilent’s DSOX2WAVEGEN 2000 X series oscilloscopes (Agilent Technologies Inc., CO, USA). Mass spectra were collected using three frequency scan modes: RF scan (boundary ejection), AC scan (resonance ejection with AC), and RF + AC scan. RF scans apply RF fields to both X and Y electrodes, while AC scans only apply an AC waveform to X electrodes. In RF + AC scans, the RF field applies to both X and Y electrodes, and the AC field applies to the X electrode. During resonance ejection, ions are ejected when their secular frequencies match the applied AC frequency^26,27^ and are detected by the CSPD. Despite the use of Faraday cage, the CSPD still picks up variable levels of noise that interfere with the ion signal. To address this, entropy-based wavelet (EBW) packet decomposition theory is employed to analyze and remove these interference components.

### Samples

Immunoglobulin G from bovine serum (IgG; MW ∼ 150 kDa), trifluoroacetic acid (TFA), and sinapic acid (SA) were purchased from Sigma (St Louis, Missouri, USA). Alpha-2-macroglobulin from human plasma (A2M; MW ∼ 362 kDa) was obtained from Athens Research and Technology (Athens, Georgia, USA). Protein samples were prepared with 5 μM concentrations. The SA matrix solution with a 2 mg/100 μL concentration was prepared in 50% acetonitrile/water (v/v) containing 0.01% TFA. The protein-matrix mixtures of IgG-SA (∼10 μL) and A2M-SA (∼10 μL) were deposited onto a stainless-steel sample probe (8 mm in diameter) for mass analysis.^17,22,28^

### Fundamentals

#### Observation Model of the Ion Signal

A model to describe the ion signal is as follows
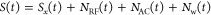
1

In eq 1, the ion signal *S*(*t*) can be decomposed as the sum of four components: *S*_*x*_(*t*) *is* the signal without noise, *N*_RF_(*t*) is the RF interference from the driving voltage of LIT, *N*_AC_(*t*) is the AC interference from the superimposed voltage from the X electrode pair of LIT, and *N*_w_(*t*) is the zero-mean Gaussian white noise. Here, we focused on the model of *N*_s_(*t*) and *N*_w_(*t*)*.* The *RF* and AC interferences, *N*_S_(*t*) in eq 2, are induced by the RF driving voltage of LIT, *V*_RF_(*t*), and AC voltage of LIT, *V*_AC_(*t*). We assume that *N*_S_(*t*) is linearly proportional to *V*_S_(*t*)
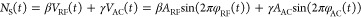
2Here, β and γ are the coefficients of sinusoidal, RF, and AC power interferences, and *A*_RF_ and *A*_AC_ are the amplitudes of these interferences. φ(*t*), φ_RF_(*t*), and φ_AC_(*t*) are the phases of sinusoidal interference, RF, and AC voltage, respectively, and they are calculated by eqs S1–S3 in Section I in the Supporting Information

The discontinuity of the waveform is observed when the frequency scan is used to execute the mass scan. Jagged edges are observed throughout the waveform, resulting in periodic spikes in the baseline intensities.^27^ Therefore, to smooth the waveform with the frequency-scan method, a phase scan φ is applied for our system with frequency changing from *f*_RF_ initial to *f*_RF_ final and from *f*_AC_ initial to *f*_AC_ final for scanning. The details of the calculation are shown in eqs S1–S7 in Section I of the Supporting Information

#### Wavelet Selection Criteria

The traditional methods^6,7,16^ of selecting a suitable wavelet for mass spectrometry involve estimating the polynomial order (M) of ion signals and choosing a wavelet with M + 1 vanishing moments. However, this approach faces five challenges in CSPD LIT-MS, where the RF frequency scan covers a broad mass range. First, some wavelets may not capture the intricate details of ion signals, causing information loss or noise interference. Second, certain wavelet families are computationally demanding, increasing processing times, especially for mass spectral forms containing multiple signal peaks. Third, this can lead to inaccuracies in signal analysis due to a failure to fully capture signal nuances. Fourth, fine-tuning wavelet parameters is complex, requiring expertise in wavelet theory. Finally, evaluating every wavelet is time-consuming. Because of those challenges, developing a novel denoising approach is necessary to solve the strong RF/AC interferences in the LIT-MS system.

This study recommends using the energy-to-Shannon entropy criterion (Section II in the Supporting Information) to select a suitable wavelet for denoising mass spectra acquired from CSPD LIT-MS. High energy concentration in a few wavelet coefficients has demonstrated the correct identification of the wavelet family by focusing on the important parts of the signal. Additionally, low Shannon entropy indicates a suitable wavelet, meaning that the signal will have a clearer structure and less complexity after transformation. The energy-to-Shannon entropy method quantifies the uncertainty or complexity of a signal by measuring its spectral entropy. In this context, Shannon entropy serves as an indicator of the complexity of the signal across both the time and frequency domains.^9,29^ The Shannon entropy of the wavelet can be calculated by associating each wavelet with a probability density function. The mother wavelet that yields the highest energy-to-Shannon entropy ratio is deemed the most suitable for signal analysis. This approach allows for the precise removal of noise while preserving the intricate details of the mass spectra, enhancing the overall accuracy and sensitivity of the LIT-MS system.

#### Wavelet Coefficient Thresholding

A clear method to identify noise components in LIT mass spectrometers is lacking. This study identifies noise in MALDI LIT-MS by minimizing energy sums across detail sub-bands up to cD1, ensuring gradual convergence to zero for RF and AC noise components, following principles outlined by Donoho and Johnstone.^30^ They have proposed setting the threshold value as a function of the median of the detailed sub-bands. Xu et al.^7^ specified selecting the cD_1_ level to filter out white noise (Gauss) components and weak-intensity sine waves.

The hard thresholding scheme is adopted in this study,^6,31,32^ and the hard thresholding filter is denoted as . In soft thresholding, wavelet coefficients are set to zero when smaller than the threshold T. For coefficients greater than T, they are shrunk toward zero. The formula for hard and soft thresholding is given by eqs S20–S22 in Section II, the Supporting Information.

## Results and Discussion

### Wavelet Selection

The wavelet transform was applied to analyze a raw signal with 200,000 data points. In dyadic wavelet packet decomposition (WPD), the theoretically deepest decomposition level J is given by log_2_(*L*), where *L* is the signal length. Since the nominal resolution of ∼10 Da^16^ was achieved over the mass range of 10 to 100 kDa, the number of detail coefficients dropped below 10 after level 13, and mass spectrum peak distortion began after level 12. Thus, decomposition up to level 11 was selected to reduce noise interference. The detailed coefficient lengths for each level are provided in Table S1.

Wavelet selection among families was optimized using the energy-to-Shannon entropy ratio (ESER) (eqs S12–S16), which balances two key aspects: energy preservation and entropy reduction. Energy preservation retains most of the signal’s energy in wavelet coefficients, while entropy reduction minimizes randomness in their distribution by reducing Shannon entropy. However, excessive entropy minimization can lead to overcompression, causing information loss. Optimizing ESER ensures efficient compression while preserving the signal’s structure. In wavelet theory,^10,13,33^ approximation coefficients capture general signal features via low-pass filtering. In contrast, detail coefficients, obtained from high-pass filtering, highlight high-frequency elements such as peaks or edges, crucial for applications such as data compression and noise reduction.

The ESER method, when used as a wavelet selection criterion, focuses on detail coefficients to isolate noise-related microstructures effectively. In this study, decomposition level *J* = 11 was selected, yielding one approximation level (cA11) and 11 detail levels (cD1–cD11), where ESER was calculated for each detail level to analyze noise concentration. Derived from 105 wavelet families, ESER values provide insights into their effectiveness in denoising mass spectra of IgG and A2M. A higher ESER value indicates a wavelet’s ability to retain spectral features while reducing noise, enhancing the signal-to-noise ratio (S/N).

**Figure 1:**
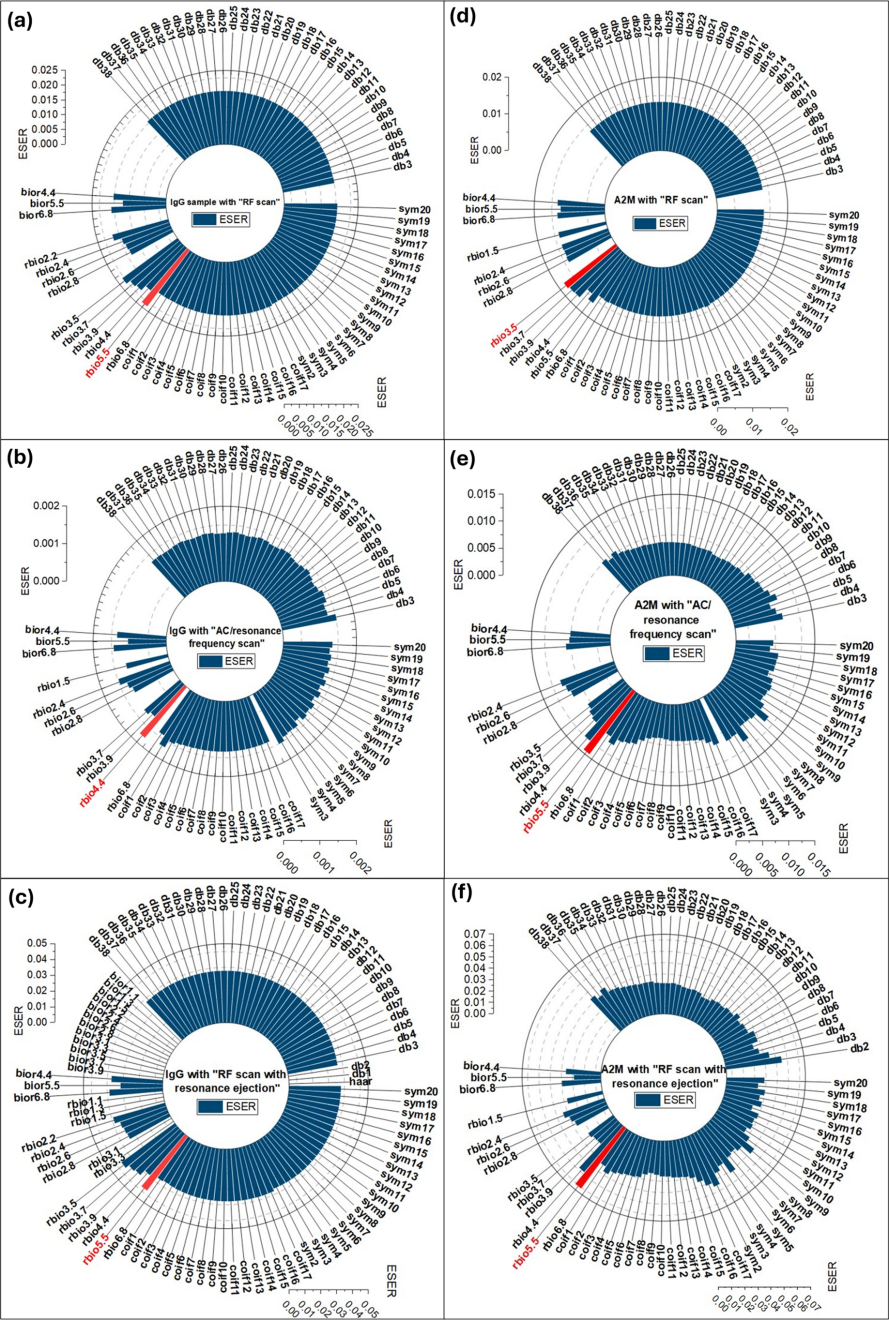
Radial bar spectra showing ESER dependence across 11 wavelet decomposition levels in MALDI LIT-MS. Red bars indicate wavelets with the highest ESER. Left column: IgG ions in (a) RF, (b) AC, and (c) RF+AC modes; right column: A2M ions in (d) RF, (e) AC, and (f) RF + AC modes.

The radial bar spectra in Figure 1 show ESER variations across 11 decomposition levels for IgG and A2M samples under three scan modes. In the RF mode, the RF voltage alone increases RF amplitude and ejects ions via boundary ejection but is inefficient, causing ion loss and poor signal quality. The AC mode applies RF voltage to the Y electrodes for ion trapping and scans the AC voltage on the X electrodes to eject ions by dipolar resonance. However, the lack of RF voltage on the X electrodes lowers the ion trapping efficiency, reducing the signal intensity. The RF + AC mode simultaneously scans both RF and AC frequencies, enhancing the ion ejection efficiency and signal clarity through resonance. The adoption of three scan modes for mass analysis helped to evaluate the method’s efficacy across various ion ejection conditions.^26^

Figure 1a–c shows ESER for IgG ions in RF, AC, and RF + AC modes, while Figure 1d–f shows ESER for A2M ions in the same modes. Red bars identify wavelet families with the highest ESER: Rbio 5.5 (RF mode), Rbio 4.4 (AC mode), and Rbio 3.5 (RF + AC mode) for IgG; Rbio 3.5 (RF mode) and Rbio 5.5 (AC and RF + AC modes) for A2M. These results highlight the importance of selecting optimal wavelet families to reduce noise and improve S/N. Since mass data are influenced by varying frequency scan ranges in CSPD LIT-MS, the choice of RF/AC waveform significantly affects ion signals. Thus, no single wavelet is universally optimal, necessitating the selection of the most suitable wavelet for effective noise reduction.

### Wavelet Packet Decomposition and Denoising of IgG and A2M Samples

**Figure 2:**
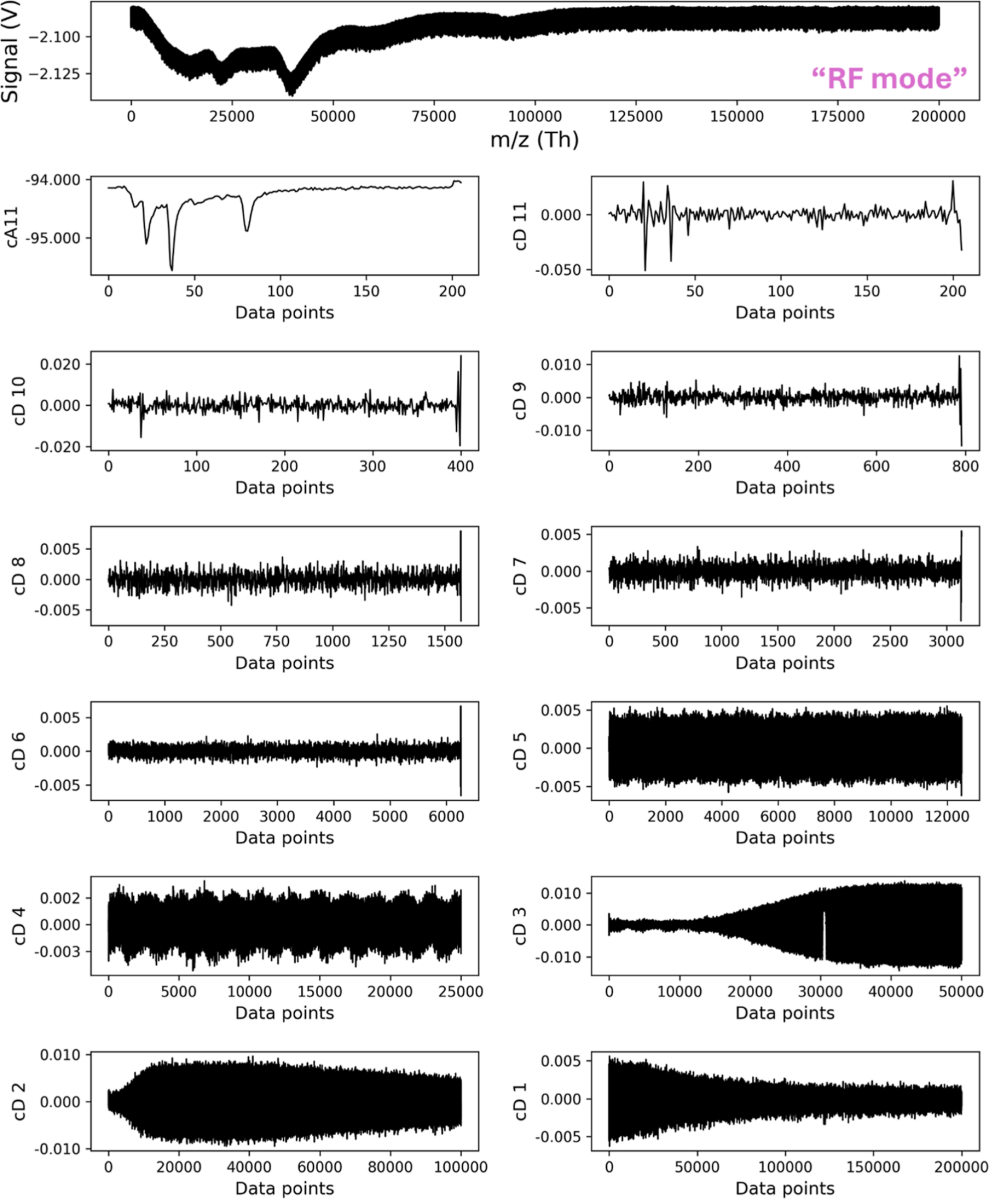
Raw signal and decomposition levels for IgG in RF mode. (a) Raw IgG spectrum; (b) decomposition using the Rbio5.5 wavelet with 1 cA11 level and 11 detail levels (cD11–cD1).

After identifying the optimal wavelet for IgG samples in all three scan modes, we decomposed the raw signal into one approximation level (cA11) and 11 detail levels (cD1–cD11) as per eqs S9 and S10. Figure 2 shows the raw mass spectrum of the IgG sample acquired in RF mode from mass 25 to 200 kDa with 200,000 data points and the IgG mass spectra after 11 decompositions. Mass spectra were obtained by applying biorthogonal wavelet 5.5 (Rbio 5.5) with one approximation level (cA11) containing 200 data points, while the ith of 11 detail levels within the range of cD1–cD11 has 200,000/2 shows the raw mass spectrum of the IgG sample acquired in RF mode from mass 25kDa to 200kDa with 200,000 data points and the IgG mass spectra after eleven decompositions. Mass spectra were obtained by applying reverse biorthogonal wavelet 5.5 (Rbio 5.5) with one approximation level (cA11) containing 200 data points, while the ith of 11 detail levels within the range of cD1 – cD11 has 200,000/2^*i*^ (where *i* = 1 to 11) data points. Detail levels cD1 and cD2 represent RF interference, while cD3 shows interference above ∼75 kDa (or ∼20,000 data points). Levels cD4–cD9 primarily contain white noise, and cD10–cD11 retain IgG signals with minimal noise.

**Figure 3:**
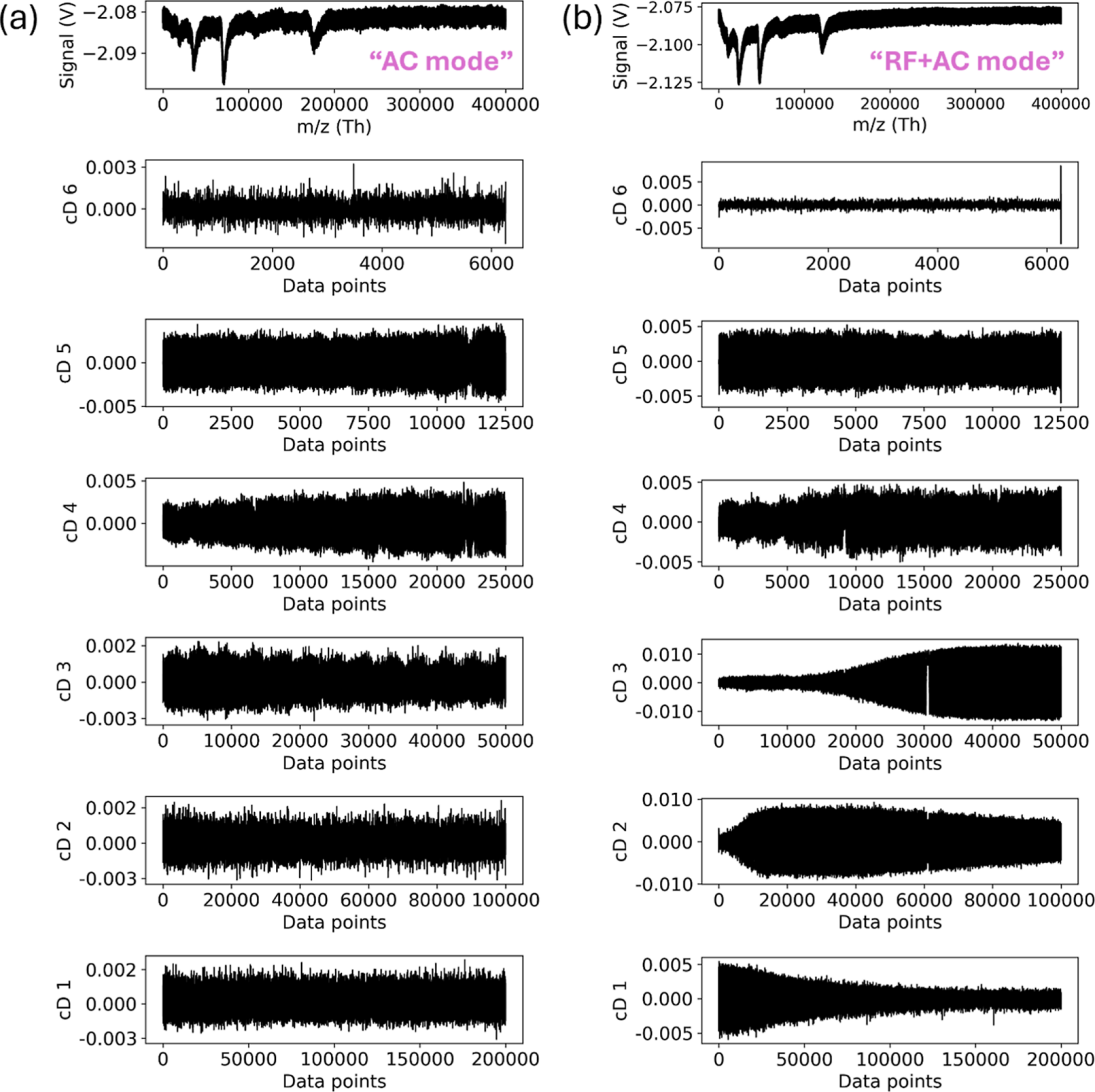
Raw signal and decomposition levels for IgG in AC and RF + AC modes. (a) Raw spectrum in AC mode; (b) decomposition in AC mode using Rbio4.4 with six detail levels (cD6–cD1); (c) raw spectrum in RF + AC mode; (d) decomposition in RF + AC mode using Rbio5.5 with six detail levels (cD6–cD1).

**Figure 4:**
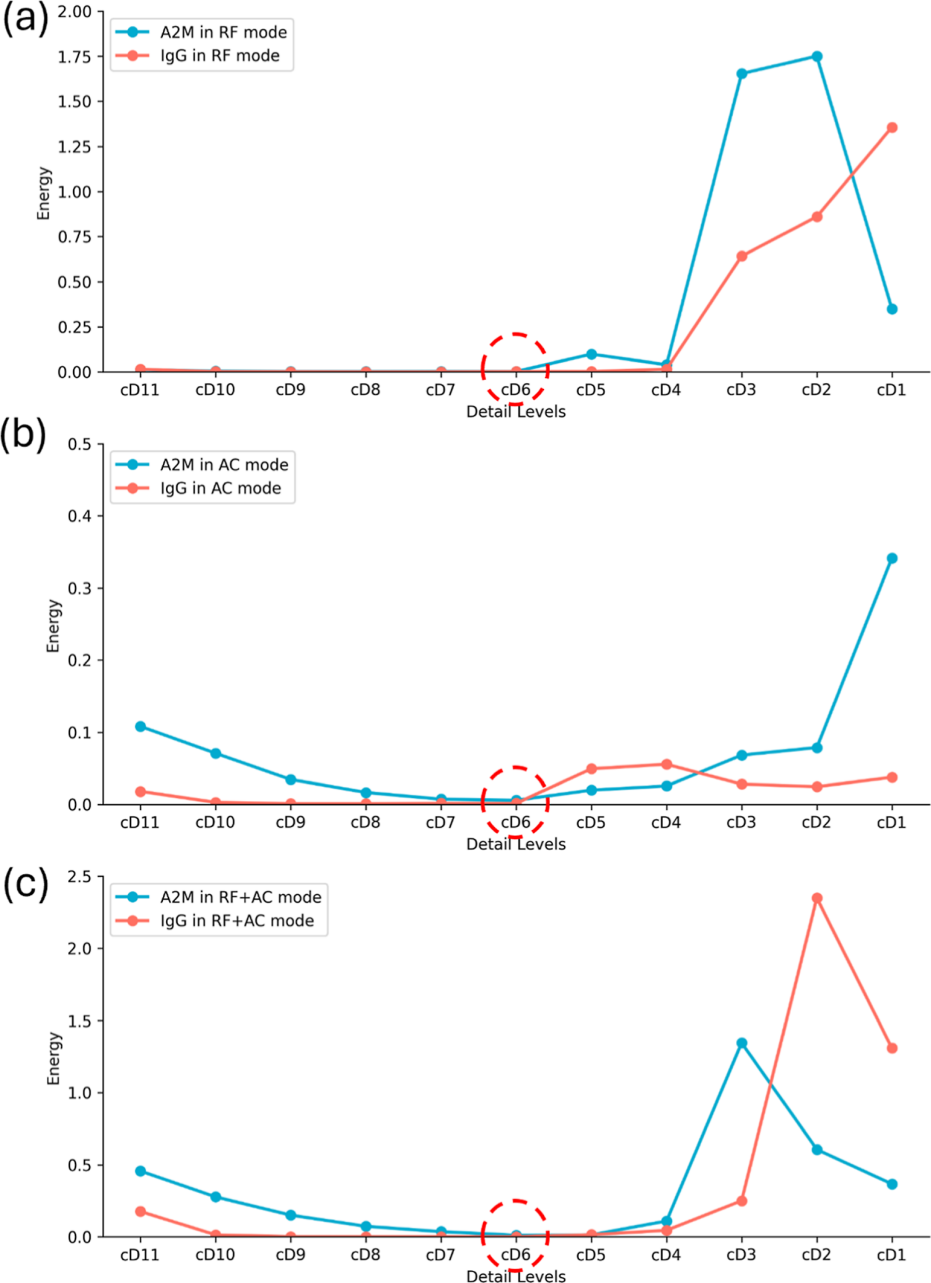
Energy distribution at decomposition levels for IgG and A2M signals in (a) RF, (b) AC, and (c) RF + AC modes.

In AC and RF + AC modes (Figure 3), Rbio 4.4 and Rbio 5.5 were used, respectively, to analyze cD1–cD6. In the RF + AC mode, cD1–cD3 resembled RF and AC modes, indicating shared noise characteristics. Noise energy is concentrated in cD1–cD3, while higher levels (cD6–cD11) are aligned with the main signal. Figure 4 confirms interference peaks in cD1–cD6, highlighting the need for noise filtering at lower levels. Applying threshold filters (eq S22) to cD1 significantly reduced noise, making it the most effective level across IgG and A2M samples for optimizing signal quality.

### Distribution of Energy in Each Level of IgG and A2M Samples

In the RF mode (Figure 4a), energy decreases from cD11 to cD8 for both IgG and A2M and then gradually rises from cD7 to cD1. A2M shows greater fluctuations, with energy dropping at cD6, steadily increasing to cD2, and slightly declining at cD1. Both samples follow a similar trend from cD11 to cD8 and increase from cD8 to cD1.

In the AC mode (Figure 4b), energy decreases from cD11 to cD8 and rises, with A2M gradually increasing from cD6 to cD1. RF + AC mode (Figure 4c) shows comparable patterns, with energy decreasing from cD11 to cD6 and steadily increasing to cD1 for both samples.

**Figure 5:**
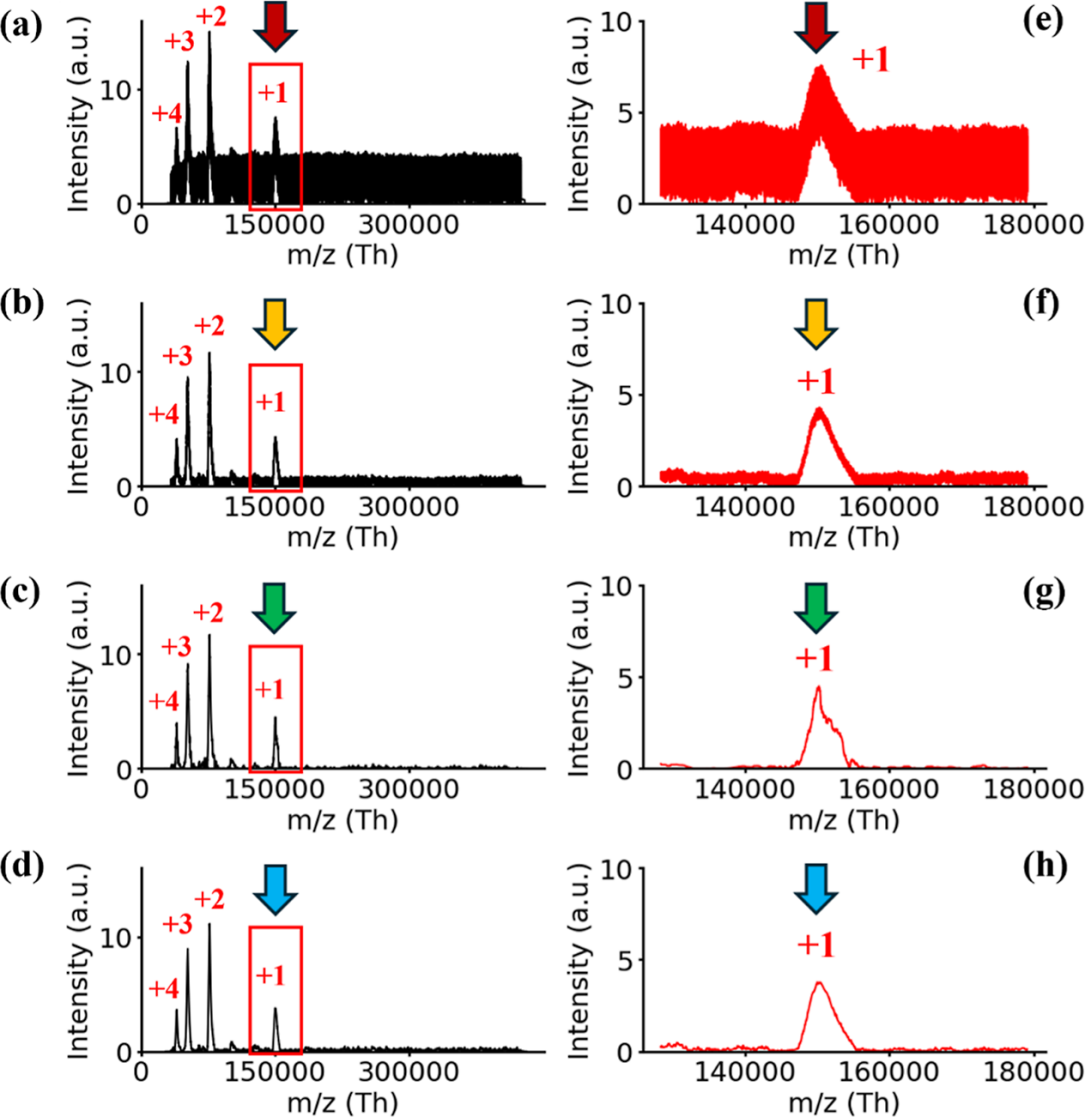
Comparison of three denoising methods for IgG in RF + AC mode: (a) raw signal; (b) wavelet-based method (‘db4’); (c) entropy-based wavelet method (11 levels); and (d) entropy-based wavelet method (6 levels). Zoomed-in IgG peaks corresponding to (a–d) are shown in (e–h).

Overall, the energy decreases from cD11 to cD6 and fluctuates upward to cD1 in all modes. A hard threshold filter (eqs S20 and S21) effectively reduced RF/AC noise while preserving key signal components (Figure 5c,d).

### Improving Signal-to-Noise by the Entropy-Based Wavelet (EBW) Method

The entropy-based wavelet (EBW) method reduces noise in CSPD LIT-MS by targeting RF/AC interference, particularly in cD1 and cD6 (Figure 4). This approach enables precise noise reduction, demonstrated with an IgG sample (Figure 5). Before calculating S/N ratios for IgG and A2M, the denoised signal undergoes charge-voltage conversion (*Q*–*V* converter, Figure S2), with results in Figure S7 (Section II). After conversion, the signal is baseline-corrected using the top-hat filter algorithm (Figure S8, Section III).

We compared the EBW method with the wavelet-based (WB) method, which is a fundamental approach for noise filtering. WB methods use wavelets like Haar and Daubechies (Db), relying on vanishing moments to identify and remove noise in wavelet transformation.^6,7,34^ Noise reduction is achieved by using hard thresholding (eqs S20 and S21). In the WB method, detail levels are shallow (D1 to D4), with the decomposed signal based on Daubechies (Db) wavelets and a blind threshold for denoising.^32^ The WB method is ineffective for denoising large data sets with high noise levels. Figure S3 also shows the inefficiency of the Fourier transform (FT). Our EBW integrates WB with an entropy-based approach, optimizing noise reduction and enhancing the signal-to-noise ratio. EBW uses adaptive filtering, automatically adjusting to signal conditions without manual intervention. This improves accuracy, sensitivity, and efficiency while reducing complexity in signal processing, making it highly applicable for mass spectrum analysis across a wide mass range.

The S/N ratio of the singly charged IgG peak improved from 3.08 in raw spectra (Figure 5a,e) to 5.87 using the wavelet-based (WB) method (Figure 5b,f) and 10.7 with EBW applied across all levels (Figure 5c,g). Using EBW at six levels, the S/N ratio increased to 9.62 (Figure 5d,h). The EBW method showed a 47.5% improvement under the RF scan with resonance ejection, reaching a 71.21% improvement across all levels (cD1–cD11) and 68% at six levels (cD1–cD6). Full-level EBW yields the highest S/N enhancement, but denoising all levels (including cD10 and cD11) suppresses low-frequency components, distorting the spectrum or shifting peak positions. EBW at six levels effectively balances the noise reduction and spectral integrity, making it the most practical approach.

**Figure 6:**
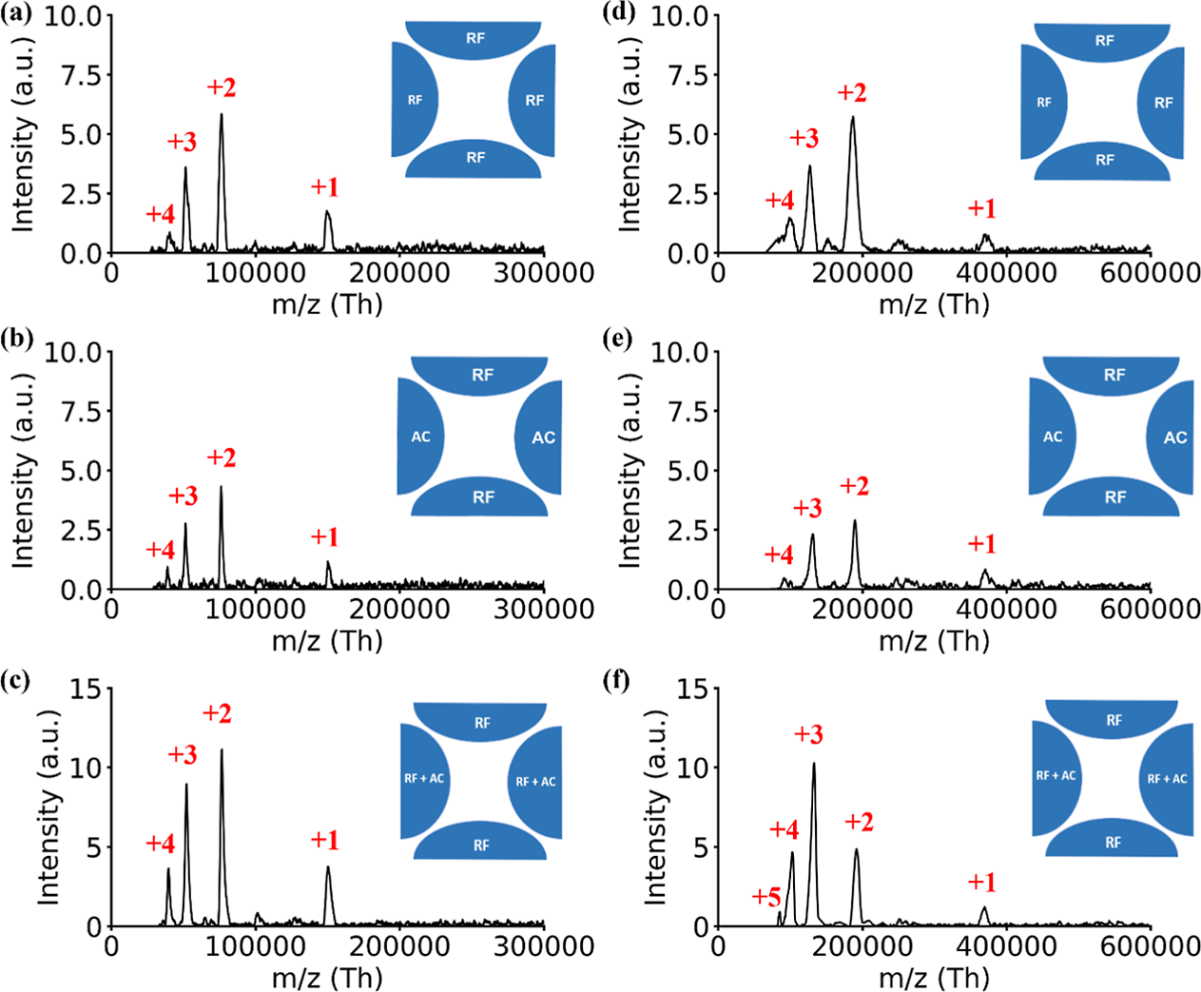
Denoised spectra of IgG (left) and A2M (right) after removing RF/AC interferences using the entropy-based wavelet (6 levels, cD1–cD6): (a) RF, (b) AC, and (c) RF + AC modes for IgG; (d) RF, (e) AC, and (f) RF + AC modes for A2M.

Figure 6 details noise reduction results for IgG and A2M samples across three scan modes, showing significant RF/AC interference mitigation. Figure 6a–c displays IgG results, while Figure 6d–f presents A2M results, illustrating interference removal across all conditions. This comparative analysis underscores the method’s robustness and adaptability, revealing consistent noise reduction across different biological samples. Details of algorithms are shown in Section IV of the Supporting Information.

To calculate the improvement of S/N from S/N_raw_ to S/N_swn_, the percentage increase is defined as eq 3 below^35^
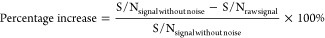
3

**Figure 7:**
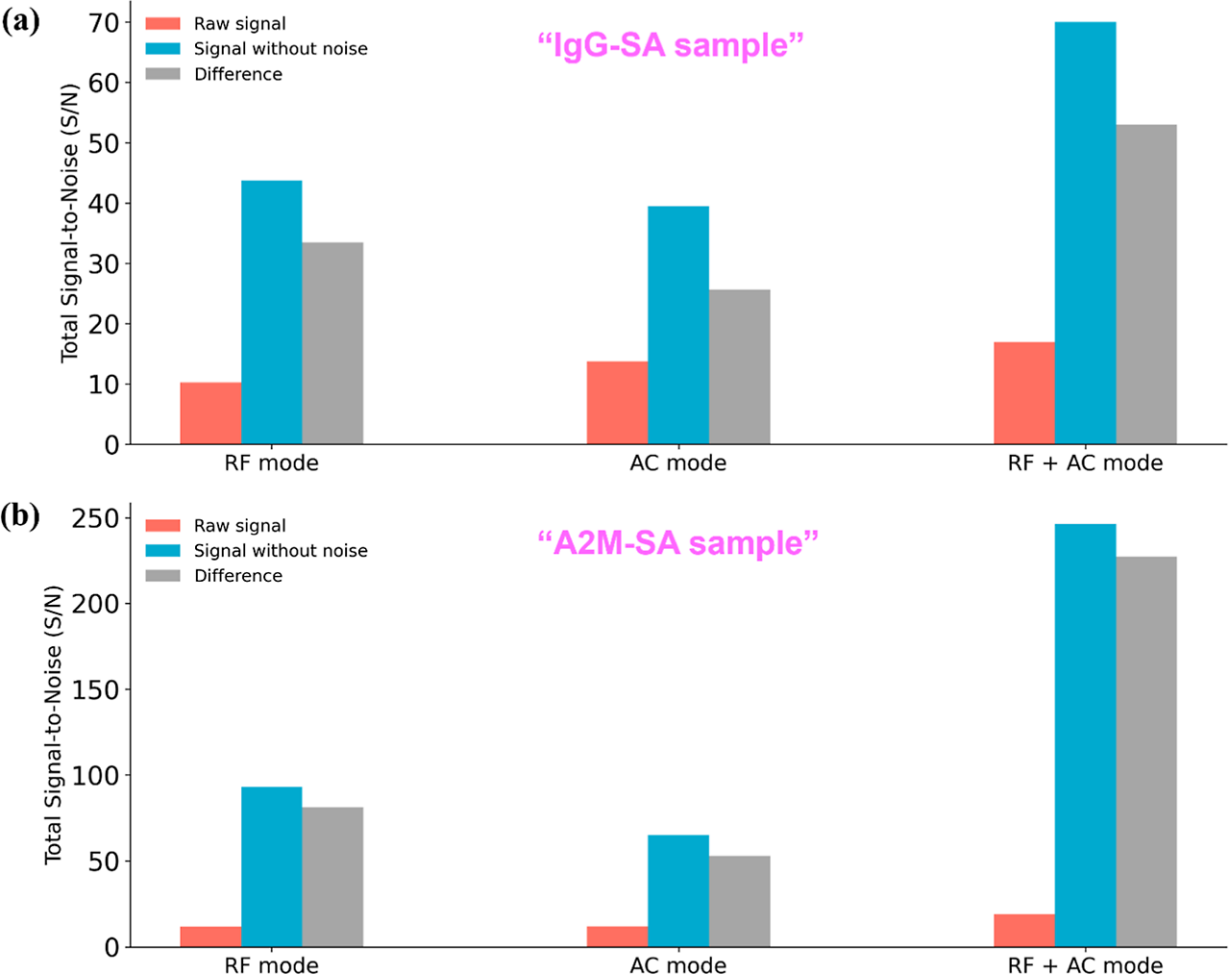
S/N ratio improvement for (a) IgG and (b) A2M spectra after RF/AC interference removal using entropy-based wavelet (6 levels, cD1–cD6) across three scan modes.

This study assessed the effectiveness of different scan modes in enhancing the S/N ratio of IgG and A2M proteins using a specific signal processing technique. Changes in S/N from the raw (S/N_raw_) to the processed value (S/N_swn_) were quantified across three scan modes: “RF mode”, “AC mode”, and “RF + AC mode”. Results were expressed as a percentage S/N increase, measured for ions of varying charge states (+1 to +5) for each protein sample (see Figure 7).

In the RF mode, the RF voltage increases the amplitude and ejects ions via boundary ejection, which leads to ion loss and poor signal quality. AC mode applies RF voltage to the Y electrode pair for trapping, scanning AC voltage on the X pair for dipolar resonance-based ejection. However, the lack of RF voltage on X electrodes reduces the trapping efficiency, lowering the signal intensity despite better ion ejection. This is evident for the +4 IgG ion and +5 A2M ion. RF + AC mode scans both frequencies simultaneously, improving ion ejection efficiency through resonance, yielding clearer, stronger signals. The results highlighted differences in S/N improvement efficiency between the two proteins, with A2M showing an average improvement (81.73%) higher than that of IgG (68.03%), suggesting variations in their chemical or physical properties. The percentage increase in S/N also varied with the charge state.

Most importantly, the S/N improvement resulted in clearer signals with reduced noise, enabling more accurate analyses. This facilitates the detection of substances at lower concentrations or obtaining more precise results. The study presents findings clearly, incorporating numerical data to enhance reliability and comparing scan modes and protein samples. Noise reduction was confirmed by comparing original and denoised S/N values under each condition, with mean S/N values for IgG and A2M summarized in Table S10.

Similar to our system, any system that relies on image charge detection techniques for ion detection and faces interferences from applied stationary and nonstationary fields could potentially benefit from our method. Charge detection mass spectrometry (CDMS)^36−39^ specifically encounters considerable difficulties in accurately detecting ions due to such noise interferences, which often mask the weaker signals from smaller or less charged ions. This issue introduces a high minimum charge requirement that exceeds the noise threshold,^40^ leading to biases toward larger or more highly charged ions. Less charged ions are typically weaker and obscured by noise, restricting the range of detectable ions^41^ and impacting overall spectrometric sensitivity and accuracy. Our entropy-based wavelet packet decomposition approach enhances the signal-to-noise ratio by focusing on the most informative signal components. It selectively isolates and amplifies crucial features, enabling the detection of ions with lower charges and masses. Moreover, its adaptive filtering dynamically adjusts to changing signal conditions, optimizing the detection process. Thus, this method could substantially improve the sensitivity and accuracy of CDMS and similar systems, reducing bias toward detecting only larger or more highly charged ions and broadening their analytical applications.

## Conclusions

We developed an entropy-based wavelet method to remove interference from strong radio frequency (RF) and auxiliary alternating current (AC) fields in a linear ion trap (LIT) mass spectrometer coupled with a charge sensing particle detector (CSPD). By optimizing the energy-to-Shannon entropy, we identified the reverse biorthogonal (RBio) wavelet family as the most effective for wavelet packet decomposition, with an optimal decomposition level of 11 for a sample length of 200,000 data points. Decomposition levels cD1-cD6 were particularly susceptible to RF/AC interference, necessitating noise reduction for accurate signal analysis. Applying a median function as a hard threshold at the cD1 level significantly reduced baseline noise, resulting in an improved signal-to-noise ratio of 68% for immunoglobulin G (IgG) ions and 82% for alpha-2-macroglobulin (A2M) ions. The presented denoising method demonstrates superior noise reduction capabilities compared to conventional techniques, enhancing the sensitivity of CSPD LIT-MS in high-mass protein analysis and offering broad applicability not only in mass spectrometry fields but also for analytical instruments operating under strong multiple RF sources.

## Data Availability

The correlation coefficient worksheet and algorithms of this article can be found as a Python package using Python code at 10.13140/RG.2.2.23488.75522 with the password of the RAR file being analytical chemistry.

## Supporting Information Available

Schematic of the charge detector linear ion trap mass spectrometer (LIT-MS), its sectional view, system operation, computer configuration, and the AcroMass charge-sensing particle detector (CSPD); Fourier transform and wavelet transform of raw ion signals; explanation of wavelet packet decomposition and wavelet selection criteria using the energy-to-Shannon entropy rate method; discussion of baseline subtraction methods, including the asymmetric least squares smoothing (ALS) algorithm, the adaptive iteratively reweighted penalized least squares (AIRPLS) algorithm, and the top-hat filter; algorithm procedures for denoising sinusoidal interference and reducing Gaussian white noise in CSPD LIT-MS, along with flowcharts; wavelet decomposition plots for an A2M sample in “RF mode”, “AC mode”, and “RF + AC mode”; *Q*–*V* conversion plots using CSPD; spectra plot after top-hat filter baseline subtraction; and energy levels after applying the entropy-based wavelet (EBW) method to denoise RF/AC interference (PDF)
