# Supplementary material for: Optimization of the Entropy-Based Wavelet Method for Removing Strong RF and AC Interferences in a Charge Detection Linear Ion Trap Mass Spectrometer
Source: Anal Chem. 2025 Feb 26;97(9):5066–76. doi: 10.1021/acs.analchem.4c06069 (PMC11912120; doi:10.1021/acs.analchem.4c06069)
Supplement: Supplementary file 1 — ac4c06069_si_001.pdf [file ac4c06069_si_001.pdf]

Supporting Information

Optimization of the Entropy-Based Wavelet Method for Removing Strong RF and AC Interferences in a Charge Detection Linear Ion Trap Mass Spectrometer

Minh Cong Dang[1,2,3], Avinash A. Patil[1], Ly Khanh Thi Lai[1], Szu-Wei Chou[1], Trang Kieu Thi Hoang[2], Mhar Ian Cua Estayan[1,4], Wen-Ping Peng[1,*]

[1]Department of Physics, National Dong Hwa University, Shoufeng, Hualien 97401, Taiwan,

[2]Department of Nuclear Physics, University of Science, Vietnam National University – Ho Chi Minh City, Ho Chi Minh City 700000, Vietnam

[3]Department of Tracer Technology, Centre for Applications of Nuclear Technique in Industry, Vietnam Atomic Energy Institute, Lam Dong 670000, Vietnam

[4]Department of Mathematics and Physics, University of Santo Tomas, Manila 1008, Philippines

* To whom correspondence should be addressed, e-mail: pengw@gms.ndhu.edu.tw

Contract/grant sponsor: NSTC NSTC 113-2112-M-259-002-, 112-2112-M-259-015-, 113-2112-M-259-006-, and 114-2923-M-259-001-MY3 (W.P.P.)-

The supporting information contains:

37 Pages, 4 Sections, 8 Figures, and 10 Tables.

Contents Sections Page

Section 1. CSPD Linear Ion Trap Mass Spectrometer Instrumentation.............................. S5

Section 2. Fourier Transform and Wavelet Transform of Raw Ion Signals S8

Section 3. Baseline Selection and Subtraction S16

Section 4. The Algorithm Procedures in Denoising Sinusoidal Interference and

Reducing Gaussian White Noise in CSPD LIT-MS S18

FIGURES

Figure S1. Schematic of CSPD linear ion trap mass spectrometer (LIT-MS) and their section view with system operation S7

Figure S2. The charge-to-voltage conversion circuit (QVC) consists of an Op Amp with a pair of common-source JFET amplifier, a picofarad capacitor parallel with a giga-ohm resistor, and a resistor network for output compensation of the Op Amp S8

Firure S3. Raw IgG ion signal and its FT frequency spectrum S10

Figure S4. Raw signal and decomposition levels of the A2M sample were obtained using RF scan mode.. S24

Figure S5. Raw signal and decomposition levels of the A2M sample were obtained using AC scan mode.. S25

Figure S6. Raw signal and decomposition levels of the A2M sample were obtained using RF+AC scan mode.. S26

Figure S7. The signal after removal is a strong RF + AC interference, and the mass spectra are converted by CSPD with configuration RF + AC mode.. S29

Figure S8. Baseline subtraction in MALDI CSPD LIT-MS with configuration RF + AC mode with Y-unit is arbitrary unit (a.u.).. S30

TABLES

Table S1. The length of detail coefficients at each level. S12

Table S2. The Mean, Median and Energy values of IgG at each detailed level with three ms-scan configurations using MALDI CSPD LIT-MS. S27

Table S3. The Mean, Median and Energy values of A2M at each detailed level with three ms-scan configurations using MALDI CSPD LIT-MS. S28

Table S4. The attenuation of a total energy at each level corresponds to the threshold setting by the coefficient sigma with the RF mode condition for the IgG sample. S31

Table S5. The attenuation of the total energy at each level corresponds to the threshold setting by the coefficient sigma with the AC mode condition for the IgG sample S32

Table S6. The attenuation of the total energy at each level corresponds to the threshold setting by the coefficient sigma with the RF + AC mode condition for the IgG sample. S33

Table S7. The attenuation of the total energy at each level corresponds to the threshold setting by the coefficient sigma with RF mode condition for the A2M sample.................................... S34

Table S8. The attenuation of the total energy at each level corresponds to the threshold setting by the coefficient sigma with AC mode condition for the A2M sample. S35

Table S9. The attenuation of the total energy at each level corresponds to the threshold setting by the coefficient sigma with the RF + AC mode condition for the A2M sample. S36

Table S10. The S/N values of IgG and A2M sample in RF mode, AC mode and RF+AC mode S36

MAIN SECTION

I. Charge Detector Linear Ion Trap Mass Spectrometer Instrumentation

To smooth the waveform with frequency-scan method, a phase scan φ is introduced[1].

$$N_S(t) = \alpha A \sin\left(2\pi\varphi(t)\right) = \beta A_{rf} \sin\left(2\pi\varphi_{rf}(t)\right) + \gamma A_{AC} \sin\left(2\pi\varphi_{AC}(t)\right) \tag{S1}$$

Where $\alpha, \beta, and\ \gamma$ are the coefficients of sinusoidal, *RF*, and *AC* power interferences. A, A_{RF}, and A_{AC} are the amplitude of the sinusoidal, RF, and AC power interferences. $\varphi(t)$, $\varphi_{rf}(t)$, and $\varphi_{AC}(t)$ are the phase of sinusoidal interference, *RF* and *AC* voltage. The change in RF/AC amplitude is referred to as a time-dependent sinusoidal function, which corresponds to the phase of the sinusoid at every time step (e.g., the i_{th} phase in the waveform array, where *i* is an integer from 0 to $\left|\frac{\tau}{\Delta t}\right| - 1$ with τ the scanning time). The relationship between the frequency *f* and phase φ is defined as [12, 24],

$$\varphi_{rf}(t_i) = f_{rf} \left|\frac{\tau}{\Delta t}\right| (t\%\Delta t) + \sum_{i=0}^{\left|\frac{\tau}{\Delta t}\right|-1} f_{(rf)i}\Delta t + \frac{\phi_{f_{(rf)i}}}{2\pi} \tag{S2}$$

$$\varphi_{AC}(t_i) = f_{AC} \left|\frac{\tau}{\Delta t}\right| (t\%\Delta t) + \sum_{i=0}^{\left|\frac{\tau}{\Delta t}\right|-1} f_{(AC)i}\Delta t + \frac{\phi f_{(AC)i}}{2\pi} \tag{S3}$$

where Δt is the duration that stays at a certain frequency, | | is the round down filter, $f_{(RF)i}$ is the stepwise sweep frequency at step *i*, $\phi_{f(RF)i}$ is the phase shift at $f_{(RF)}$, which can be estimated by tone analysis according to the read back value from LIT driving voltage, $f_{(AC)i}$ is the stepwise sweep frequency at step *i*, $\phi_{f(AC)i}$ is the phase shift at $f_{(AC)i}$ which can be estimated by tone analysis

according to the read back value from LIT scanning voltage, and % indicates the modular. The number of steps is set as *K*, and the frequency of linear frequency stepwise scan is defined[12] as

$$f_{(rf)i} = \frac{i}{K}\left(f_{rf\ final} - f_{rf\ initial} \right) + f_{rf\ initial} \tag{S4}$$

$$f_{(AC)i} = \frac{i}{K}\left(f_{AC\ final} - f_{AC\ initial} \right) + f_{AC\ initial} \tag{S5}$$

Where $f_{RFinitial}$, $f_{RFfinal}$, $f_{ACinitial}$ and $f_{ACfinal}$ are the *RF* initial frequency, the *RF* final frequency, the *AC* initial frequency, and the *AC* final frequency.

The relationship between the *RF* and the *AC* frequency is,

$$\frac{d\theta_\omega}{d\theta_\Omega} = \frac{\beta.\beta_{scale}}{2} \text{ where } \begin{cases} \dfrac{d\theta_\Omega}{dt} = \text{ frequency of } rf \text{ waveform} \\ \dfrac{d\theta_\omega}{dt} = \text{ frequency of } AC \text{ waveform} \end{cases} \tag{S6}$$

$$\begin{aligned} &\Rightarrow \frac{f_{AC\ initial}}{f_{rf\ initial}} = \frac{f_{AC\ final}}{f_{rf\ final}} = \frac{\beta.\beta_{scale}}{2} \\ &\Rightarrow \begin{cases} f_{AC\ initial} = \dfrac{\beta.\beta_{scale}}{2} f_{rf\ initial} \\ f_{AC\ final} = \dfrac{\beta.\beta_{scale}}{2} f_{rf\ final} \end{cases} \end{aligned} \tag{S7}$$

Where θ_ω is the cycle of the *AC* waveform, θ_Ω is the cycle of the *rf* waveform, and β is the beta value used to define the auxiliary *AC* frequency and the corresponding m/z value of mass spectra. β_{scale} defines the ratio between *AC* frequency and *rf* frequency.

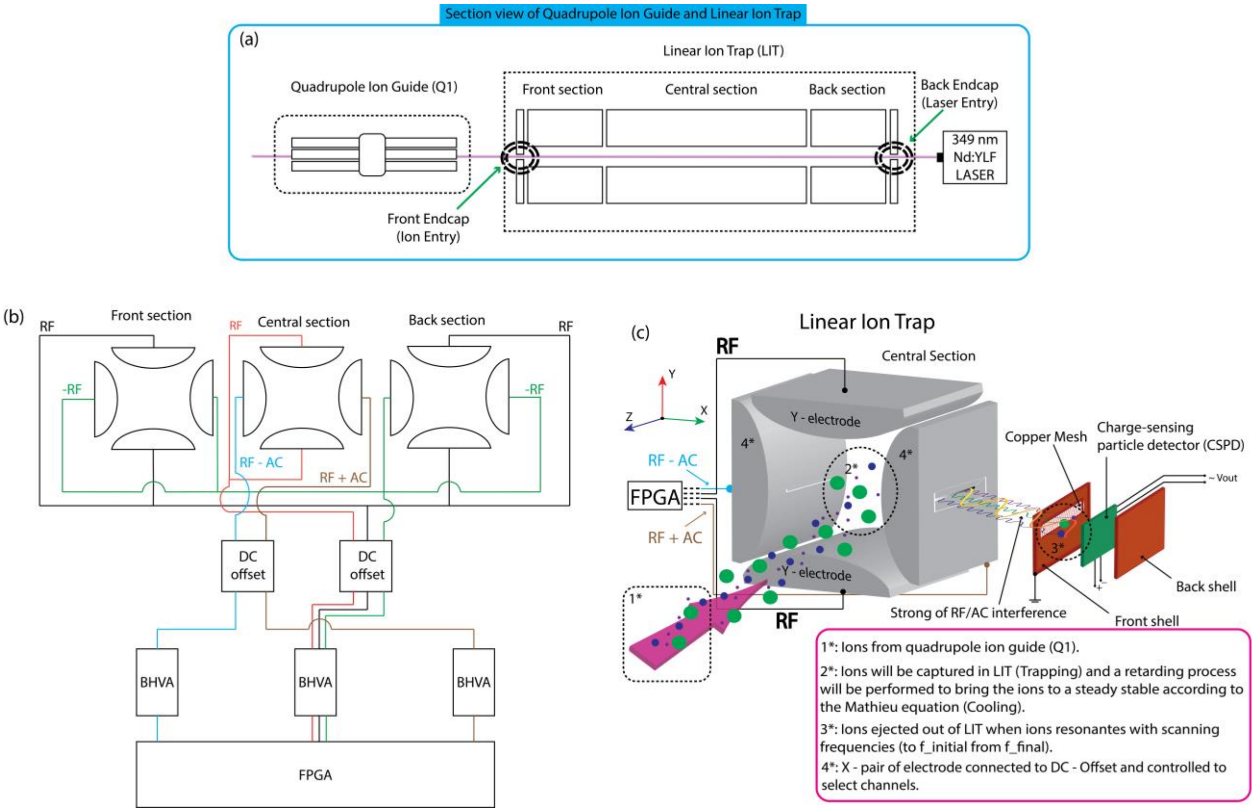

Figure S1. Schematic of CSPD linear ion trap mass spectrometer (LIT-MS) and their section view with system operation.

Algorithm Development

The data are processed by ATI software (for Windows 10 - AcroMass Technologies Inc, Taipei, Taiwan, Republic of China) with the computer using a 12th Gen Intel(R) Core (TM) i9-12900K (3.20GHz) with a 31.7 GB RAM. Algorithms are written by Python 3.9.0 (Jupyter, ver. 2022.11.1003262421 for Windows 10) with an AMD Ryzen 5 2500U (2.00GHz) computer with 6.95 GB RAM.

Charge-Sensing Particle Detector (CSPD)

Charge-sensing particle detector (CSPD) senses the current variation of incoming ion packet and represents it as a pulse signal. The CSPD is composed of a copper mesh, an integrated circuit board (ICB), a charge detection plate (Faraday tray), an integrated circuit (IC) unit, and an interference shielding unit (Faraday cage). The charge detection plate is located on the front of the double-sided ICB, with the IC unit electrically connected and positioned on the non-coplanar back side for shielding from incoming ion packets. The interference shielding unit reduces RF and AC field interference from the Linear Ion Trap (LIT) and features a nickel mesh (98% transmission) above the detection region to manage incoming ions.

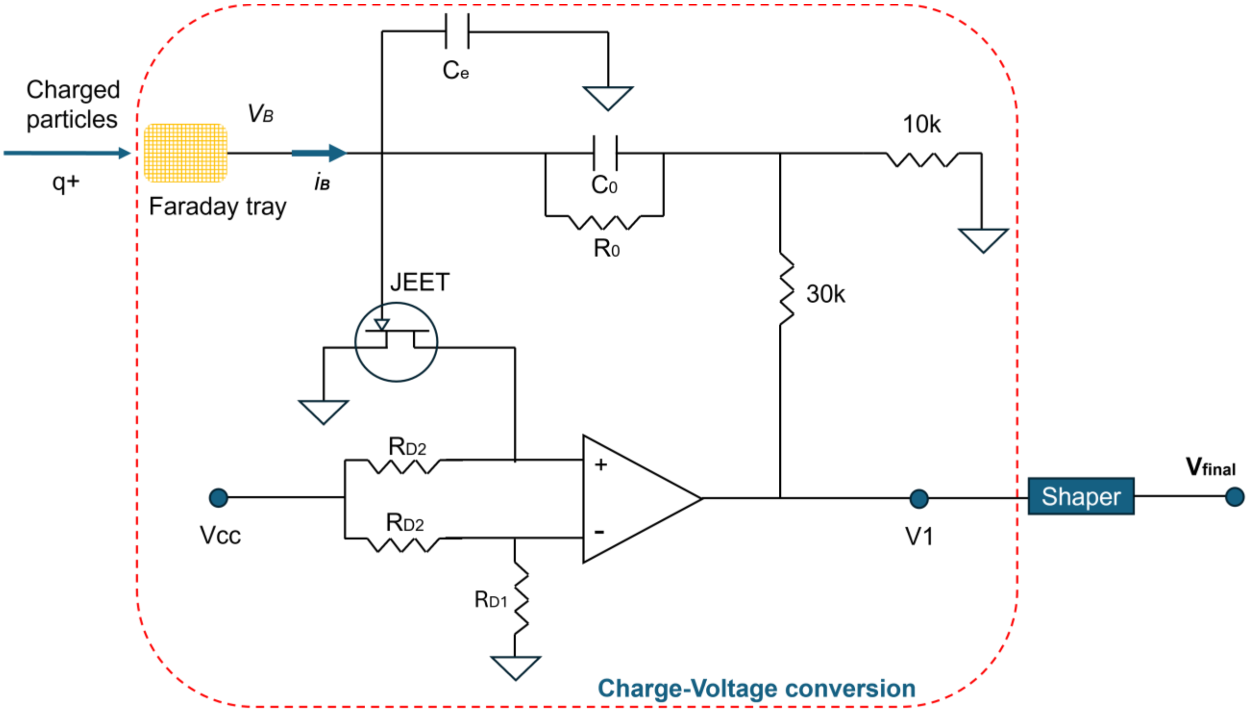

Figure S2. The charge-to-voltage conversion circuit (QVC) consists of an Op Amp with a pair of common-source JFET amplifier, a picofarad capacitor parallel with a giga-ohm resistor, and a resistor network for output compensation of the Op Amp[2].

II. Fourier Transform and Wavelet Transform of Raw Ion Signals

Fourier Transform

Fourier Transform (FT) Method: FT is a traditional signal analysis tool that transforms signals from the time domain to the frequency domain[3, 4]. However, it assumes signals are stationary and cannot provide localized time information regarding to its frequency components. This limits its effectiveness in handling non-stationary or nonlinear signals, particularly in mass spectrometry applications with time-varying noise.

$$F(f) = \int_{-\infty}^{\infty} x(t) e^{-j2\pi ft} dt \tag{S8}$$

F(f) is the function in the frequency domain (Fourier spectrum), *x(t)* is the function in the time domain (original signal), *f* is the frequency (Hz), *t* is time, *j* is the imaginary unit (j = −1), and $e^{-j2\pi ft}$ is the complex kernel of the Fourier transform.

We present an example in Figure S3 to evaluate the performance of the FT method using an IgG sample acquired in RF mode for signal analysis. Figure S3a shows the raw IgG signal, while Figure S3b displays its FT spectrum. It is evident that the frequency domain contains numerous small signals, as highlighted in the zoomed-in section of the figure. These signals and noise points are generated from the RF mass scan. This observation highlights a key challenge: using the FT method to denoise signals with time-varying or uncertain frequencies is highly likely to result in omissions or insufficient denoising. The lack of clear evidence to determine appropriate lower or

upper cut-off frequencies further complicates the process, reducing the effectiveness of FT-based denoising.

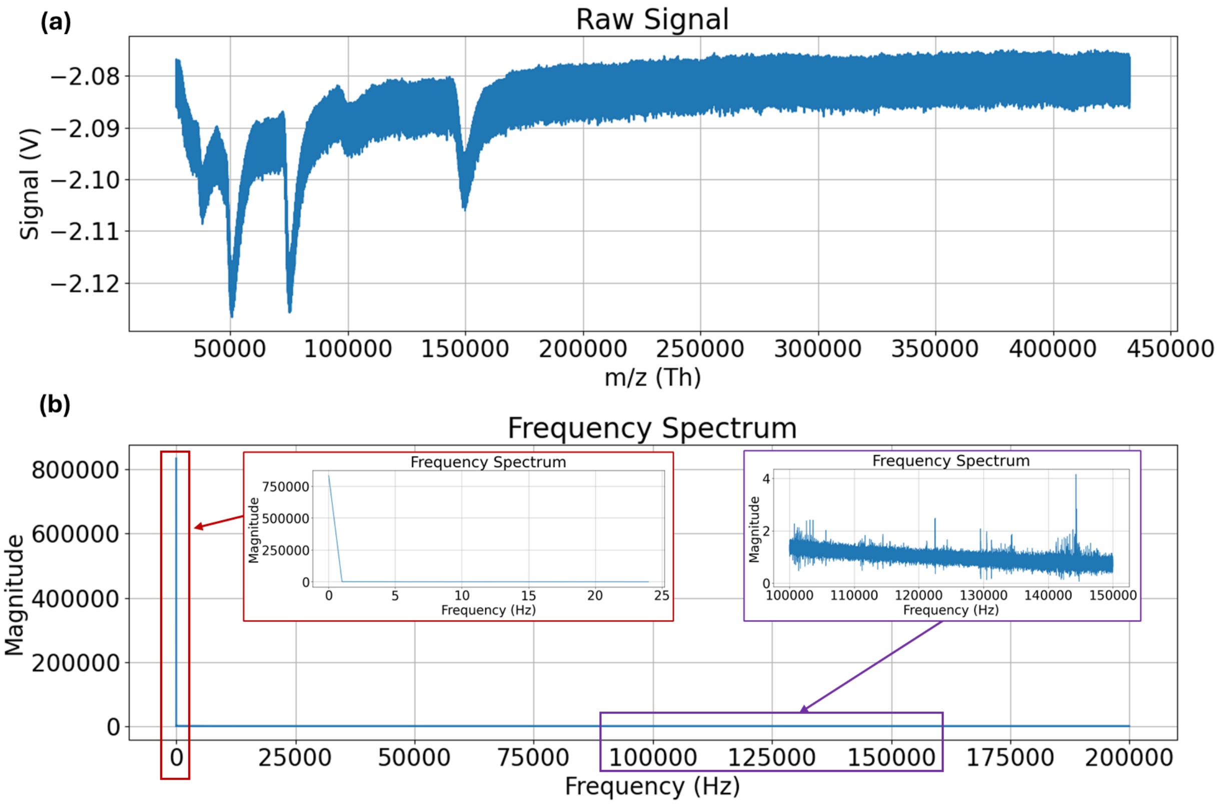

Figure S3. Raw IgG ion signal and its FT frequency spectrum. (a) raw signal of IgG sample in RF mode, (b) frequency spectrum using FT method.

Wavelet Transform

Discrete wavelet transformation is an effective time-frequency analysis method. However, it only partially decomposes the low-frequency components, and the high-frequency components are not processed, leading to poor resolution. The wavelet packet decomposition, however, handles the undecomposed high-frequency portion. This improves the resolution in time and frequency domains and can be treated more effective [5-9].

The decomposition is described as:

$$cD_{j+1}^{2p}[k]=\sum_{n=-\infty}^{+\infty}h\left[n-2k\right]cD_j^P \ and \ cD_{j+1}^{2p+1}[k]=\sum_{n=-\infty}^{+\infty}g\left[n-2k\right]cD_j^P \quad \text{(S9)}$$

The reconstruction is described as:

$$cD_j^p[k]=\sum_{n=-\infty}^{+\infty}h[k-2n]\,cD_{j+1}^{2p}[n]\oplus\sum_{n=-\infty}^{+\infty}g[k-2n]\,cD_{j+1}^{2p+1}[n] \quad \text{(S10)}$$

Where $cD_j^{2p}\left[k\right]$ and $cD_j^{2p+1}\left[k\right]$ are approximate coefficients and detail coefficients, $h[n,k]$ and $g[n,k]$ are low-pass filter and high-pass filter. The coefficients of wavelet packet children cD_{j+1}^{2p} and cD_{j+1}^{2p+1} are obtained by subsampling the convolutions cD_j^p with h and g. By iterating these equations along the branches of a wavelet packet tree, we compute all wavelet packet coefficients from the wavelet packet coefficients $\{j_i, p_i\}_{1\le i\le I}$ of a wavelet packet tree.

Wavelet packet decomposition is an effective tool for signal sequence decomposition. In mass spectrometry, we use the frequency-scan method to acquire the ion signals, the strong RF amplitude can generate significant sinusoidal interference that affects the CSPD detector. Using wavelet packet decomposition can be an effective tool to decompose the signal, i.e., separating the true signal and the corresponding noise components. The LIT is made of three sections: the front section, the central section, and the back section. In the central section, we set three ms-scan configurations by FPGA control system for X–pair electrodes, including “RF mode”, “AC mode,” and “RF + AC mode” for two samples, i.e., IgG and A2M. The volume of both IgG and A2M is 5μL and the volume of the SA matrix is ~ 10 μL, mixing with a ratio of 1:2. The mass spectrum from ATI software (Acromass company) will be converted to a .csv file and using Python 3.0 to

analyze and plot. Images of the decomposition of the A2M raw signal to approximate levels and detailed levels are shown in Fig. S3 to S5. The statistical values of three ms-scan configurations, such as the Mean, Median, and Energy (energy in data processing) to identify components noise in MALDI LIT-MS, have been specified in Table S2 and Table S3.

Table S1. The length of detail coefficients at each level.

**Level (J)**	**Length of detail coefficients**
cD_{01}	100,000 – 200,000
cD_{02}	50,000 – 100,000
cD_{03}	25,000 – 50,000
cD_{04}	12,500 – 25,000
cD_{05}	6,250 – 12,500
cD_{06}	3,125 – 6,250
cD_{07}	1,562 – 3,125
cD_{08}	781 – 1,562
cD_{09}	390 – 781
cD_{10}	195 – 390
cD_{11}	97 – 195

Wavelet Selection Criteria

Energy:

The energy content of a signal can also be calculated from its wavelet coefficients at different levels [10]. Energy is defined as:

$$E_{energy}(s) = \sum_{i=1}^{N} \left| wt(s,i) \right|^2 \tag{S11}$$

where N is the number of wavelet coefficients, and $wt(s,i)$ represents the wavelet coefficients in scale.

Shannon Entropy:

For the same amount of energy within a sub-frequency band, the condition of the signal may be different, e.g., only several frequency components with high magnitude and others with negligible magnitude over a widespread spectrum. The spectral distribution (or concentration) of the energy needs to be considered to ensure effective feature extraction with boundary conditions as defined in equations S14 and S15. Different wavelet decomposition levels should exhibit significantly change amplitude and energy, and the distribution of wavelet coefficients across different scales over the entire time domain is required to decompose the raw signal using discrete wavelet transform effectively. The energy distribution of the wavelet coefficients can be quantitatively described by the Shannon entropy [11-13]:

$$E_{entropy}(s) = -\sum_{i=1}^{N} p_i.\log_2 p_i \tag{S12}$$

where p_i is the energy probability distribution of the wavelet coefficients, defined as:

$$p_i = \frac{\left| wt(s,i) \right|^2}{E_{energy}(s)} \tag{S13}$$

with $\sum_{i=1}^{N} p_i = 1$, and $p_i \log_2 p_i = 0$ if $p_i = 0$ (S14)

Equations (S5) and (S6) indicate that the entropy of the wavelet coefficients is bound by:

$$0 \le E_{entropy}(s) \le \log_2 N \quad \text{(S15)}$$

Energy-to-Shannon Entropy Ratio (ESER) is defined as:

$$R(s) = \frac{E_{energy}(s)}{E_{entropy}(s)} \quad \text{(S16)}$$

Where *R(s)* is the energy-to-Shannon entropy ratio, $E_{energy}(s)$ is the energy of scale coefficients, and $E_{entropy}(s)$ is Shannon entropy of scale coefficients [10].

Statistical Parameters

$$\text{Mean} = \frac{\sum_{i=1}^{N} cD_{i,j}}{N},\ j < \log_2 N \quad \text{(S17)}$$

$$\text{Median} = \begin{cases} cD_{\left[\frac{i+1}{2}\right],j} & \text{if } i \text{ is odd} \\ \dfrac{cD_{\left[\frac{i}{2}\right],j} + cD_{\left[\frac{i}{2}+1\right],j}}{2} & \text{if } i \text{ is even} \end{cases},\ j < \log_2 N \quad \text{(S18)}$$

$$\text{Energy} = \sum_{i=1}^{N} \left(cD_{i,j}\right)^2,\ j < \log_2 N \quad \text{(S19)}$$

Where $cD_{i,j}$ is the detail coefficients, *j* is the decomposition level, and *N* is the length of the signal. After decomposing the raw ion signal and calculating the scaling level to 11, the energy of

detail coefficients at each level can be used to distinguish the white noise and the strong RF and AC interferences, as shown in Fig. S4 – S6.

Wavelet Coefficient Thresholding

The formula for hard and soft thresholding is given by eq. S20:

$$\tilde{T}_{hard}(cD_{i,j}) = \begin{cases} cD_{i,j}, & \text{if } |cD_{i,j}| > T \\ 0, & \text{if } |cD_{i,j}| \le T \end{cases}$$

$$\tilde{T}_{soft}\left(cD_{i,j}\right) = \begin{cases} \text{sign}(cD_{i,j}).\left(|cD_{i,j}| - T\right), & \text{if } |cD_{i,j}| \ge T \\ 0, & \text{if } |cD_{i,j}| \le T \end{cases}, \quad \text{sign}(cD_{i,j}) = \begin{cases} 1, \text{if } cD_{i,j} > 0 \\ 0, \text{if } cD_{i,j} = 0 \\ -1, \text{if } cD_{i,j} < 0 \end{cases} \tag{S20}$$

Where $\{T_j\}_{1 \le j < J}$ is the threshold at each j level, and sign($cD_{i,\,j}$) denotes the sign function of $cD_{i,j}$. Although the soft thresholding method can effectively reduce noise and produce smoother signals, it has a drawback because it diminishes the values of coefficients that exceed the threshold, as these coefficients are shrunk towards zero. This can lead to a partial loss of information in the stronger signal components. Therefore, we use hard thresholding in this study to denoise strong RF/AC interferences.

$$T_{j(hard)} = \{\sigma_j\}_{1 \le j < J} \sqrt{2\log_e N} = \frac{1}{0.6475} Med\left(cDi, j\right)\sqrt{2\log_e N} \tag{S21}$$

The standard deviation of the detail coefficient $\{\sigma_j\}_{1 \le j < J} = Med(cDi, j)/0.6475$ and Med(cDi,j) is the absolute median value normalized with the fine-scale detail coefficients11, and N is the length of the raw signal. A hard threshold (as described in eq. S21) is applied to denoise the details coefficients at each level. Detail coefficients with values exceeding this threshold will be retained, while those below the threshold will be set to zero. Based on the detailed sub-

coefficients at each level, the determination of noise components presented at each decomposition level, i.e., median and energy at each detailed sub-coefficient, could be calculated by eqs. S17 – S19 in Section II in the supporting information.

The median value of cD1 from the hard thresholding filter (eq. 4) was utilized for noise filtering in the energy distribution at each level. The hard thresholding of detail coefficients at each level j can be achieved by

$$D_{i,j}^{2p+1}[k] = \widetilde{T}\left(D_{i,j}^{2p+1}[k]\right) \tag{S22}$$

Where k ∈ Z (Z is the integers), $D_{i,j}^{2p+1}[k]$ denotes the threshold. Finally, the signal can be reconstructed in eq. 9 from $D_j^{p*}[k] = \left[\left\{D_{i,j}^{2p+1}[k]\right\}_{1 \le j < J}, D_{i,J}^{2p}[k]\right]$ through a recursive function.

III. Baseline Selection and Subtraction

Effective background subtraction can be achieved through the use of data processing software such as Origin, which provides several built-in functions for this purpose. However, these functions primarily rely on the precise identification of the peak baseline, which might not be useful when the baseline is a complex function, or the function is not easy to define. Eilers et al.[14] introduced the Asymmetric Least Squares Smoothing (ALS) algorithm, which is a widely adopted method for baseline subtraction. Zhang et al.[15] later developed the Adaptive Iteratively Reweighted Penalized Least Squares (AIRPLS) algorithm, offering improvements in adaptability and precision. Additionally, Stanford et al.[16] proposed using the Top-Hat filter for baseline subtraction of mass spectrometry data, which has shown significant improvements in handling complex mass spectra.

However, each of these algorithms has its strengths and limitations. While algorithms such as ALS and AIRPLS are known for their speed and efficiency, their main drawback lies in handling exceptionally large datasets, such as the one in our case, which exceeds 200,000 data points. These algorithms are less suitable for such large-scale data due to computational challenges. On the other hand, the Top-Hat filter algorithm, as presented by Stanford et al.[16], offers a tailored approach. This method estimates the baseline width for each peak in the mass spectrum and systematically searches for values that allow for accurate background subtraction.

The primary limitation of the Top-Hat filter is that it is computationally intensive and time-consuming, particularly when it is used to process data with standard personal computing setups. Despite this, the algorithm's ability to oversee large datasets precisely makes it the most suitable choice for our needs. Its accuracy in isolating the baseline from large and complex datasets outweighs the longer computation time. For these reasons, we have adopted the Top-Hat filter algorithm for background subtraction in our IgG and A2M mass spectra data analysis.

Compared to other methods, the Top-Hat algorithm offers numerous advantages in baseline correction for mass spectrometry. Firstly, Top-Hat effectively oversees nonlinear, complex, and highly variable baselines, which can be challenging for polynomial methods or ALS algorithms. Additionally, Top-Hat preserves the shape and height of signal peaks, particularly for sharp and narrow peaks, thus avoiding the attenuation of important signal intensities. Furthermore, the ability to customize the structuring element in Top-Hat provides flexibility, allowing for optimization of processing data based on their characteristics. Due to these advantages, Top-Hat is an ideal tool for processing complex mass spectrometry data, surpassing traditional methods.

After applying the baseline subtraction, the results can be seen in Figure S7. Figure S7 shows two mass spectra illustrating the process of baseline correction and signal analysis. Graphs (a) and (b), respectively, display the converted spectrum (black line), the estimated baseline (red line), and the baseline-subtracted spectrum (blue line). The x-axis represents the mass-to-charge ratio (m/z) in Thomson (Th), while the y-axis shows the signal intensity. The main signal peaks are labeled with red numbers corresponding to charge states such as +1, +2, +3, +4, and +5, indicating ions at different ionization states. Baseline subtraction clarifies the ion peaks, enhancing the accuracy of mass spectrometric analysis.

IV. The Algorithm Procedures in Denoising Sinusoidal Interference and Reducing Gaussian White Noise in CSPD LIT-MS

In summary, the sinusoidal interference and white noise can be removed from the signal observations raw signal by implementing the following steps:

i. Select a wavelet suitable for the input signal by the maximum values of the ESER among 105 wavelet families.

ii. Select the deepest decomposition level J*, where J corresponds to a length of detail coefficients of approximately 10^{1}.

iii. Decompose the raw signal down to level J* at step ii using equation S1.

iv. Estimate the standard deviations and obtain the thresholds that are depend on the level J in step *ii*.

v. Threshold the detail coefficients according to equation 13 in the main article and obtain the thresholded coefficients.

vi. Reconstruct the signal from the thresholded detail coefficients and the approximate coefficients according to equation S2.

vii. Convert the reconstructed signal from a charge-voltage conversion circuit (Q-V converter).

viii. Use a Top-Hat filter to subtract the baseline signal after conversion.

[1] In addition, when selecting the deepest decomposition level, J depends on boundary conditions, i.e., the length of raw signal ($0 < J < \log_2 N$) and the nominal resolution ($0 < J \leq J^*$).

Algorithm Flowcharts

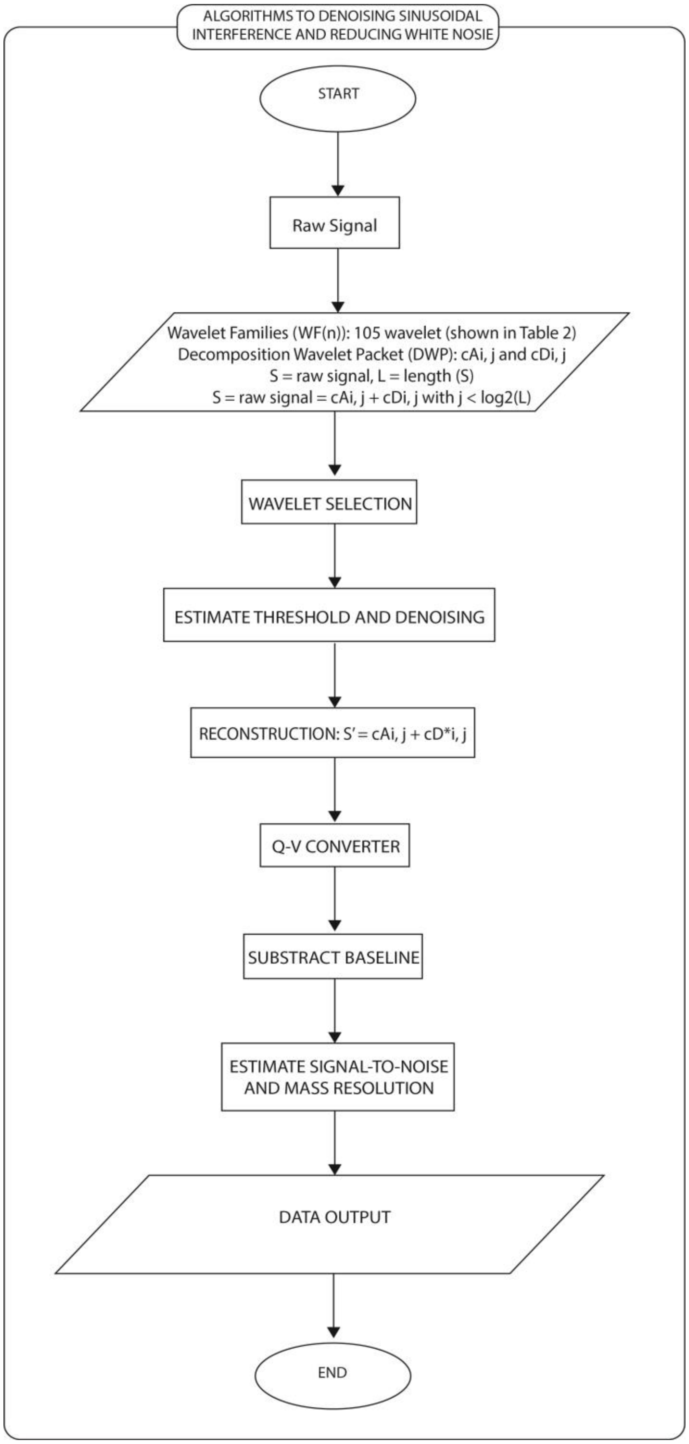

ALGORITHMS TO DENOISING SINUSOIDAL INTERFERENCE AND REDUCING WHITE NOSIE
START
Raw Signal
Wavelet Families (WF(n)): 105 wavelet (shown in Table 2)
Decomposition Wavelet Packet (DWP): cAi, j and cDi, j
S = raw signal, L = length (S)
S = raw signal = cAi, j + cDi, j with j < log2(L)
WAVELET SELECTION
ESTIMATE THRESHOLD AND DENOISING
RECONSTRUCTION: S' = cAi, j + cD*i, j
Q-V CONVERTER
SUBSTRACT BASELINE
ESTIMATE SIGNAL-TO-NOISE AND MASS RESOLUTION
DATA OUTPUT
END

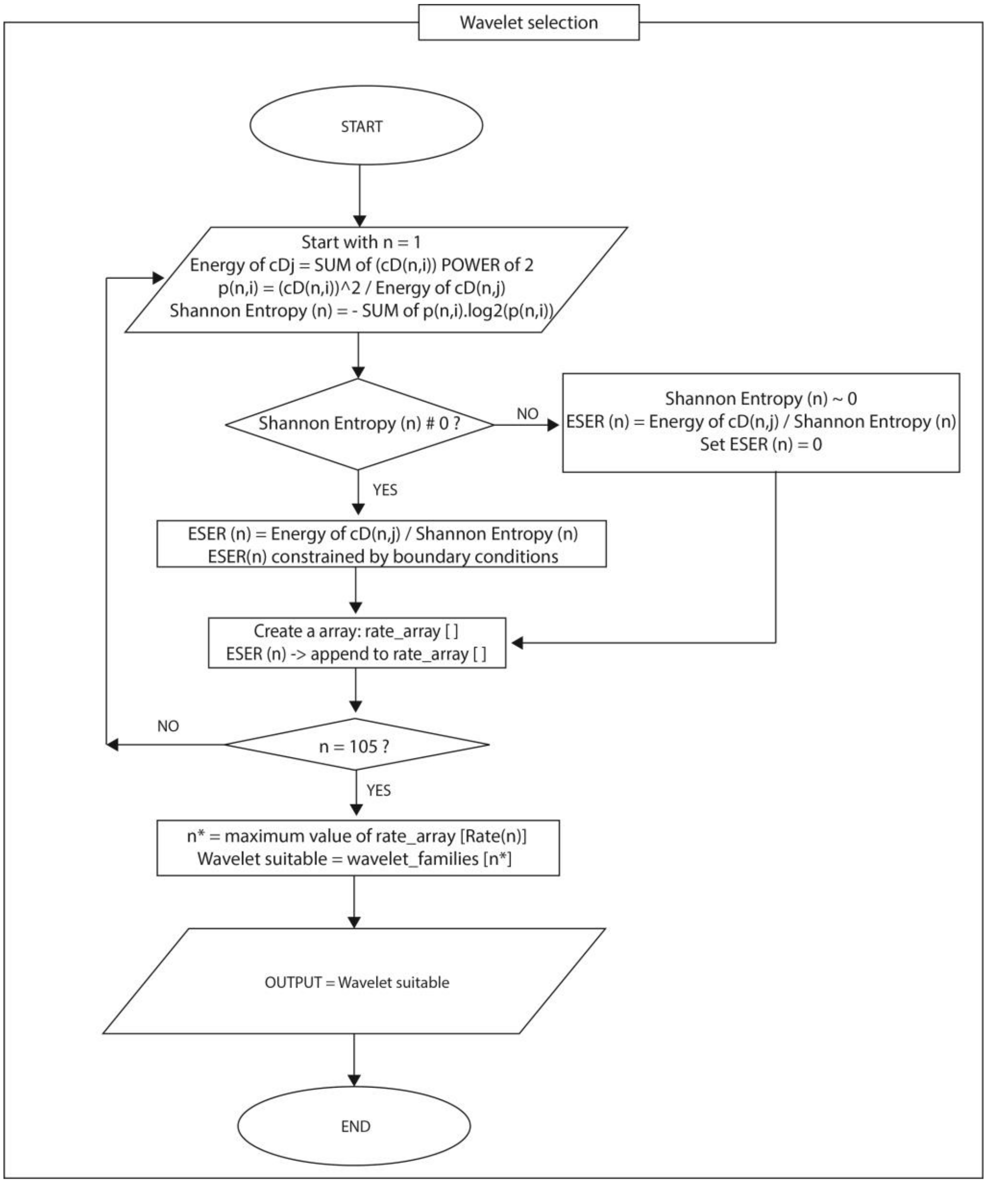
Wavelet selection
START
Start with n = 1
Energy of cDj = SUM of (cD(n,i)) POWER of 2
p(n,i) = (cD(n,i))^2 / Energy of cD(n,j)
Shannon Entropy (n) = - SUM of p(n,i).log2(p(n,i))
Shannon Entropy (n) # 0 ?
NO
Shannon Entropy (n) ~ 0
ESER (n) = Energy of cD(n,j) / Shannon Entropy (n)
Set ESER (n) = 0
YES
ESER (n) = Energy of cD(n,j) / Shannon Entropy (n)
ESER(n) constrained by boundary conditions
Create a array: rate_array [ ]
ESER (n) -> append to rate_array [ ]
NO
n = 105 ?
YES
n* = maximum value of rate_array [Rate(n)]
Wavelet suitable = wavelet_families [n*]
OUTPUT = Wavelet suitable
END

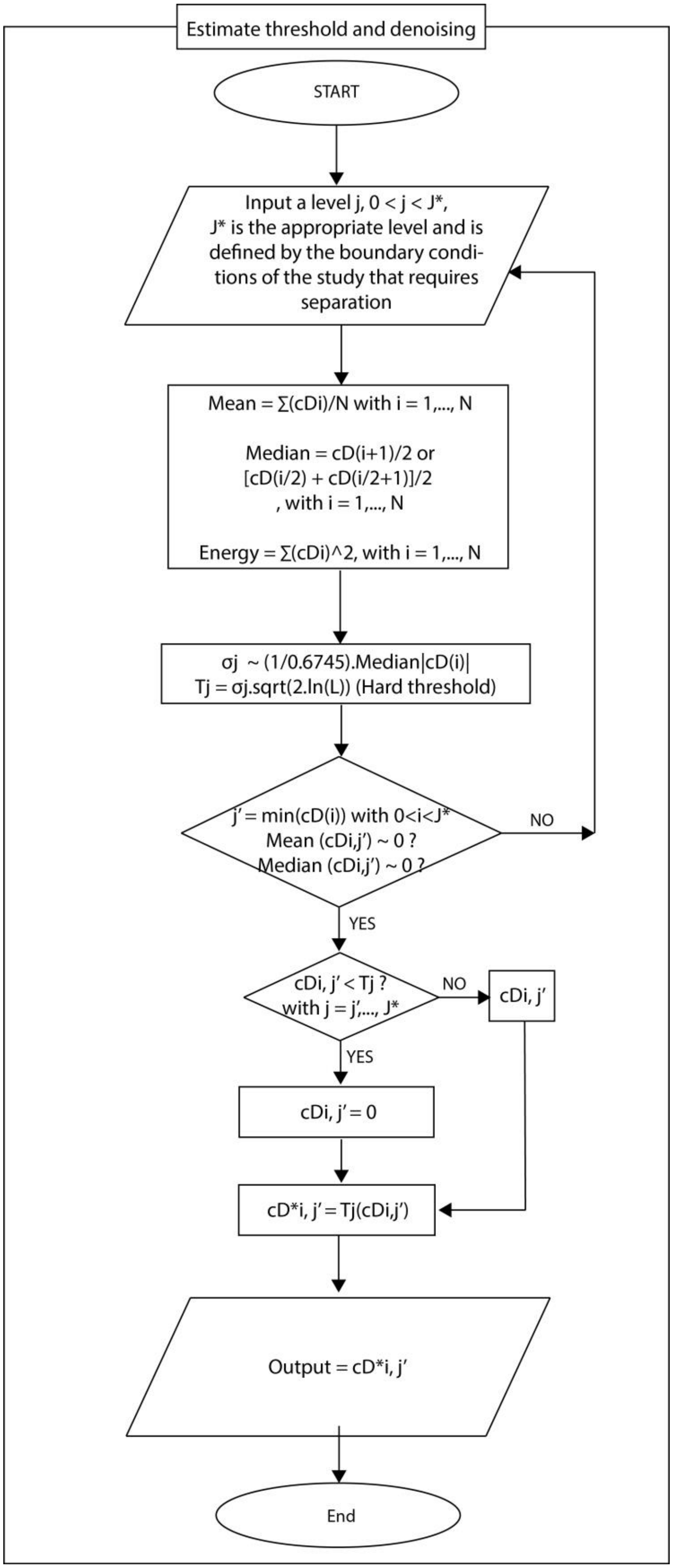
Estimate threshold and denoising
START
Input a level j, 0 < j < J*,
J* is the appropriate level and is defined by the boundary conditions of the study that requires separation
Mean = ∑(cDi)/N with i = 1,..., N
Median = cD(i+1)/2 or [cD(i/2) + cD(i/2+1)]/2 , with i = 1,..., N
Energy = ∑(cDi)^2, with i = 1,..., N
σj ~ (1/0.6745).Median|cD(i)|
Tj = σj.sqrt(2.ln(L)) (Hard threshold)
j' = min(cD(i)) with 0<i<J*
Mean (cDi,j') ~ 0 ?
Median (cDi,j') ~ 0 ?
NO
YES
cDi, j' < Tj ?
with j = j',..., J*
NO
cDi, j'
YES
cDi, j' = 0
cD*i, j' = Tj(cDi,j')
Output = cD*i, j'
End

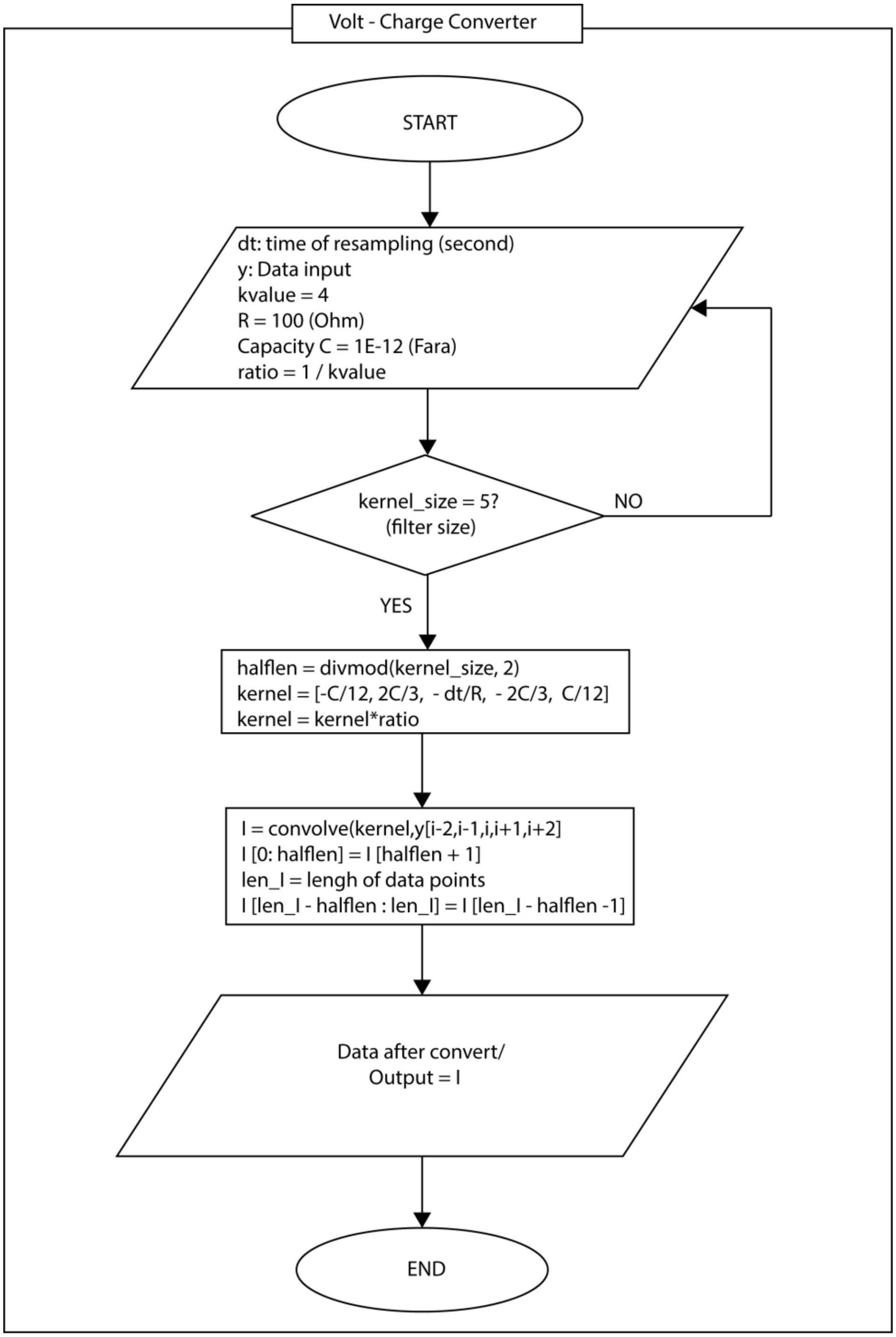
Volt - Charge Converter
START
dt: time of resampling (second)
y: Data input
kvalue = 4
R = 100 (Ohm)
Capacity C = 1E-12 (Fara)
ratio = 1 / kvalue
kernel_size = 5?
(filter size)
NO
YES
halflen = divmod(kernel_size, 2)
kernel = [-C/12, 2C/3, - dt/R, - 2C/3, C/12]
kernel = kernel*ratio
I = convolve(kernel,y[i-2,i-1,i,i+1,i+2]
I [0: halflen] = I [halflen + 1]
len_I = lengh of data points
I [len_I - halflen : len_I] = I [len_I - halflen -1]
Data after convert/
Output = I
END

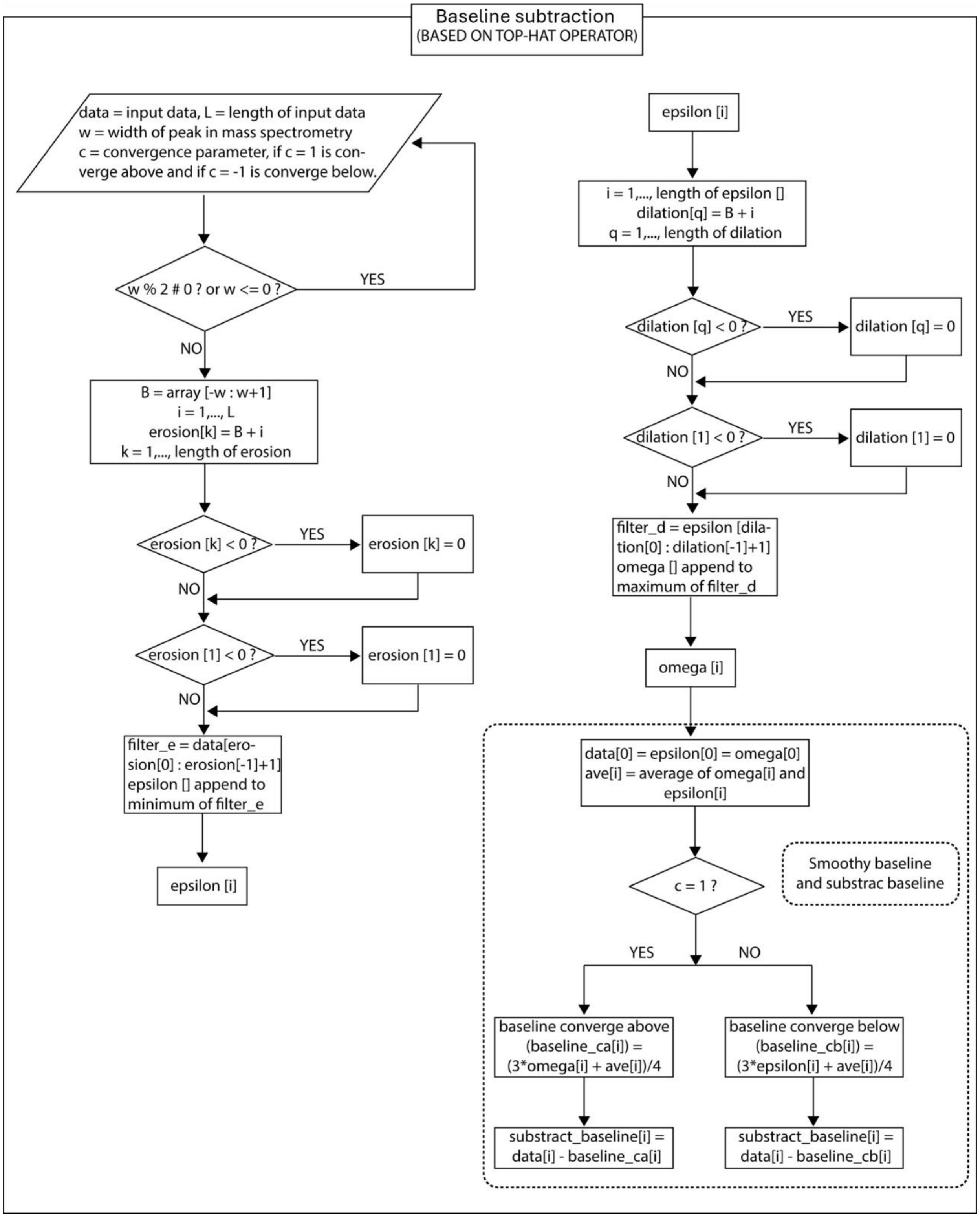
Baseline subtraction
(BASED ON TOP-HAT OPERATOR)
data = input data, L = length of input data
w = width of peak in mass spectrometry
c = convergence parameter, if c = 1 is converge above and if c = -1 is converge below.
w % 2 # 0 ? or w <= 0 ?
YES
NO
B = array [-w : w+1]
i = 1,..., L
erosion[k] = B + i
k = 1,..., length of erosion
erosion [k] < 0 ?
YES
erosion [k] = 0
NO
erosion [1] < 0 ?
YES
erosion [1] = 0
NO
filter_e = data[erosion[0] : erosion[-1]+1]
epsilon [] append to minimum of filter_e
epsilon [i]
epsilon [i]
i = 1,..., length of epsilon []
dilation[q] = B + i
q = 1,..., length of dilation
dilation [q] < 0 ?
YES
dilation [q] = 0
NO
dilation [1] < 0 ?
YES
dilation [1] = 0
NO
filter_d = epsilon [dilation[0] : dilation[-1]+1]
omega [] append to maximum of filter_d
omega [i]
data[0] = epsilon[0] = omega[0]
ave[i] = average of omega[i] and epsilon[i]
c = 1 ?
Smoothy baseline and substrac baseline
YES
NO
baseline converge above (baseline_ca[i]) = (3*omega[i] + ave[i])/4
baseline converge below (baseline_cb[i]) = (3*epsilon[i] + ave[i])/4
substract_baseline[i] = data[i] - baseline_ca[i]
substract_baseline[i] = data[i] - baseline_cb[i]

Wavelet Packet Decomposition of A2M Sample in All Modes

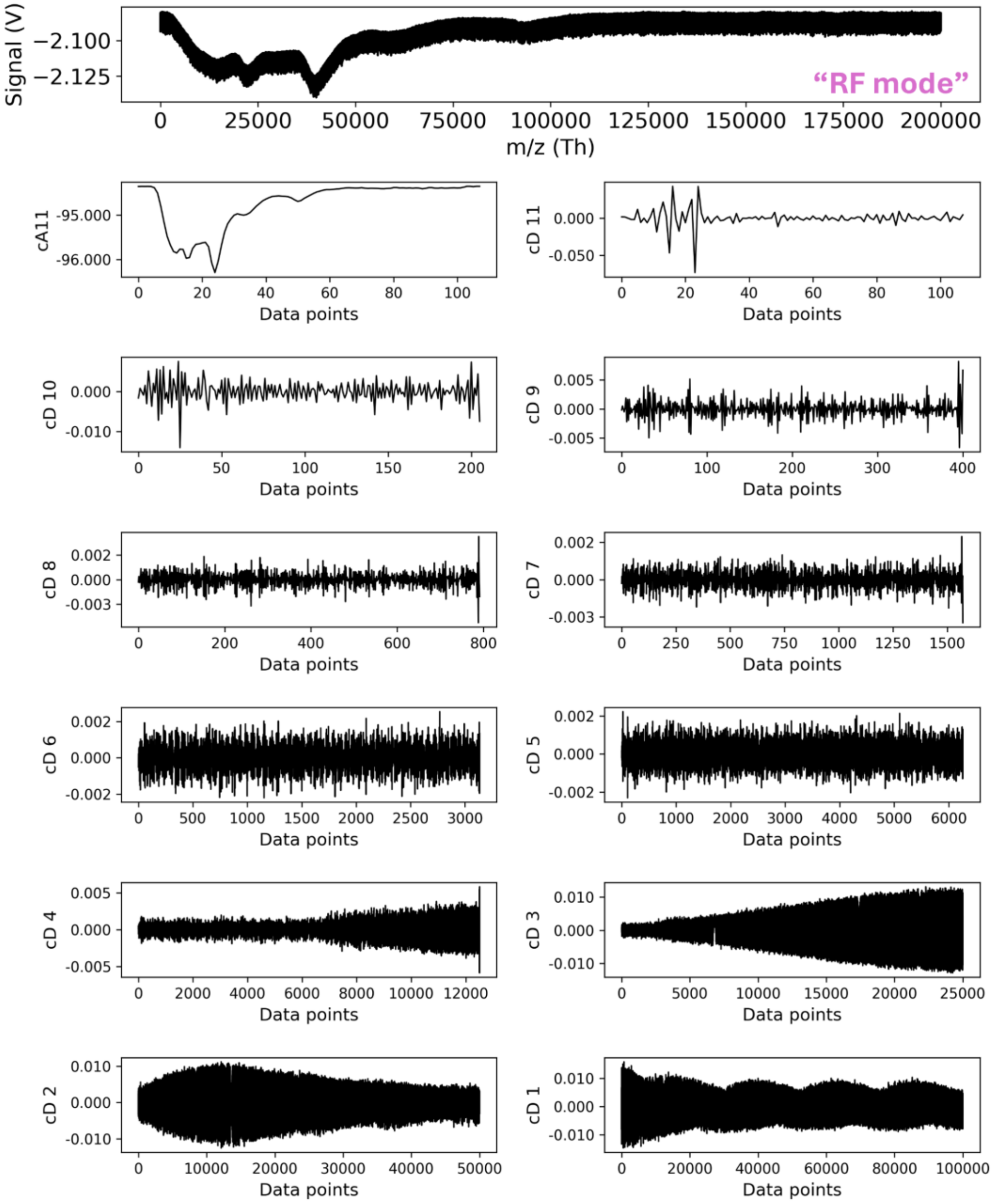

Figure S4. Raw signal and decomposition levels of the A2M sample were obtained using RF scan mode. a) Raw A2M mass spectrum b) Decomposition levels of A2M mass spectrum obtained using the reserve bi-orthogonal (Rbio3.5) of wavelet families with 01 level cA11 and detail decomposition 11 levels (cD1 – cD11).

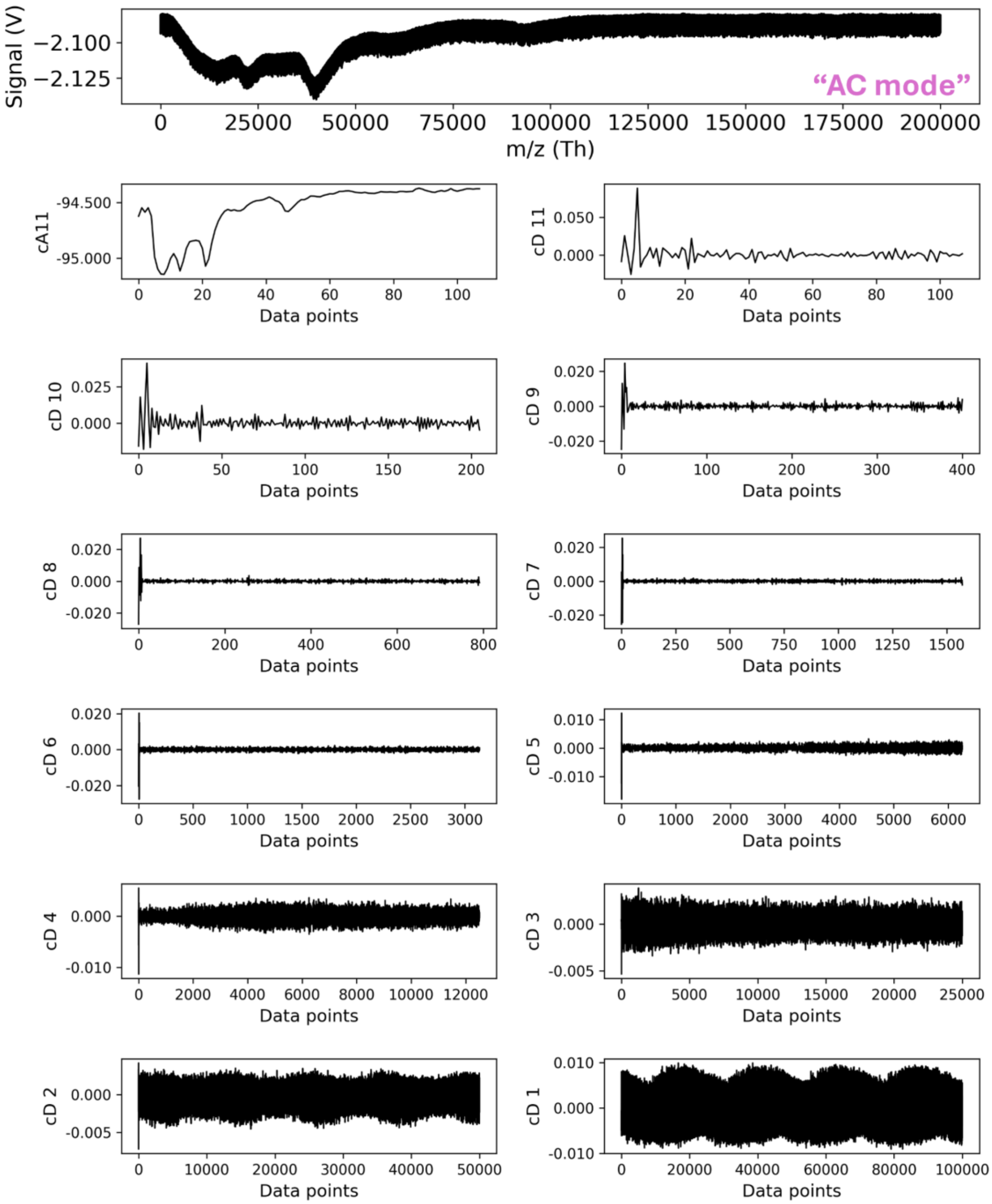

Figure S5. Raw signal and decomposition levels of the A2M sample were obtained using AC scan mode. a) Raw A2M mass spectrum b) Decomposition levels of A2M mass spectrum obtained using the reserve bi-orthogonal (Rbio5.5) of wavelet families with 01 level cA11 and detail decomposition 11 levels (cD1 – cD11).

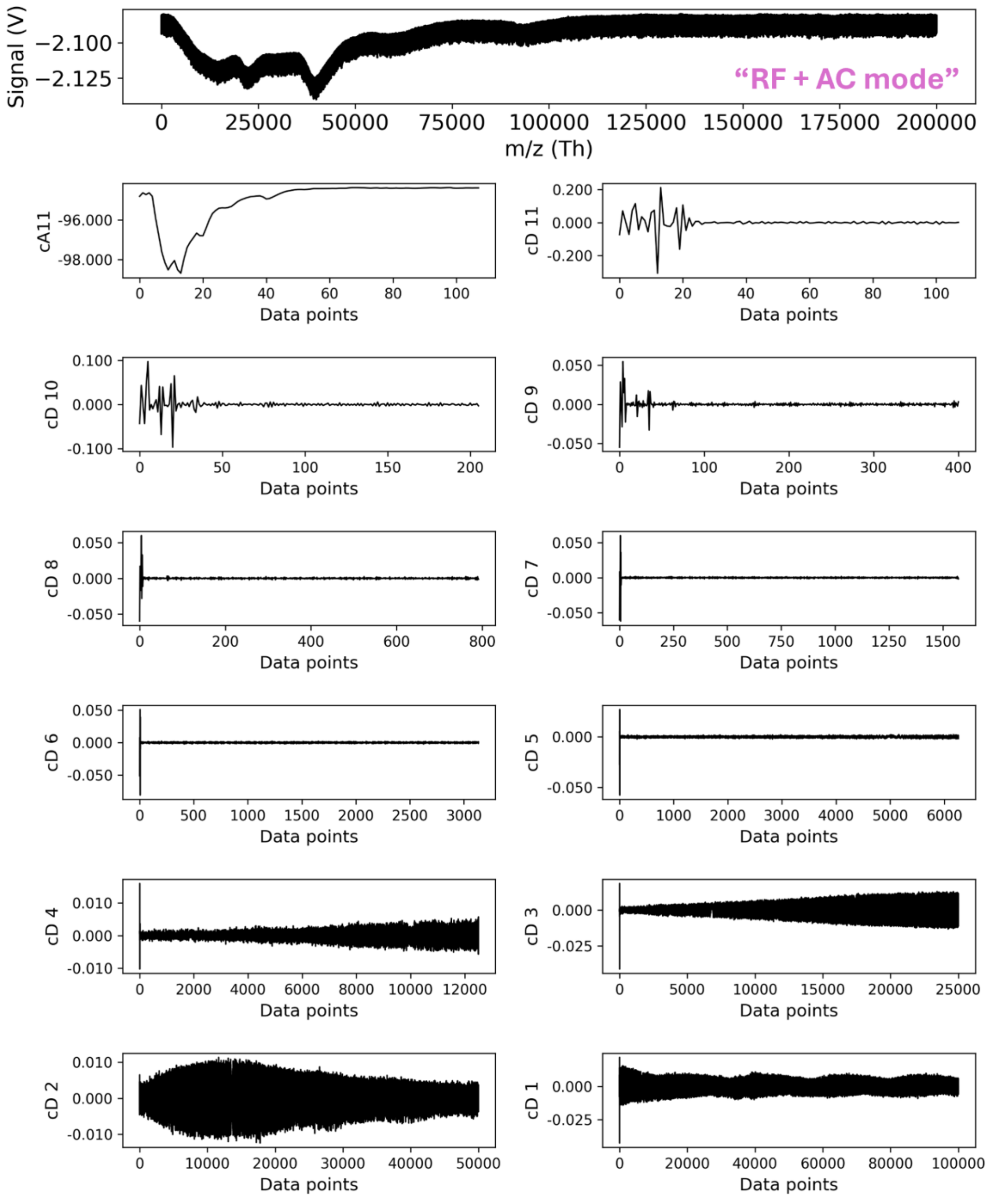

Figure S6. Raw signal and decomposition levels of the A2M sample were obtained using RF+AC scan mode. a) Raw A2M mass spectrum b) Decomposition levels of A2M mass spectrum obtained using the reserve bi-orthogonal (Rbio5.5) of wavelet families with 01 level cA11 and detail decomposition 11 levels (cD1 – cD11).

Table S2. The Mean, Median and Energy values of IgG at each detailed level with three ms-scan configurations using MALDI CSPD LIT-MS.

Detail level	MALDI LIT-MS								
	CENTRAL SECTION								
	RF mode			AC mode			RF + AC mode		
	Mean	Median	Energy	Mean	Median	Energy	Mean	Median	Energy
cD_{11}	9.58E-05	3.39E-04	1.48E-02	-2.40E-04	-2.87E-04	1.79E-02	-5.87E-05	2.84E-04	1.76E-01
cD_{10}	5.01E-05	7.08E-05	1.53E-03	-5.78E-05	-2.92E-05	2.82E-03	2.35E-05	-1.65E-05	1.36E-02
cD_{09}	-2.37E-06	1.50E-04	1.1E-03	4.68E-05	4.81E-06	1.21E-03	-8.02E-06	1.36E-05	1.54E-03
cD_{08}	-5.62E-06	2.89E-05	4.91E-04	1.02E-05	1.83E-05	1.16E-03	-7.41E-06	6.89E-06	1.04E-03
cD_{07}	-1.09E-05	-2.49E-05	5.46E-04	-1.92E-05	-4.56E-05	1.54E-03	7.40E-07	5.02E-06	1.28E-03
cD_{06}	-1.79E-06	3.11E-05	1.66E-03	-5.13E-06	-6.89E-06	1.42E-03	-3.55E-06	-7.32E-06	9.80E-04
cD_{05}	1.45E-06	2.95E-05	2.29E-03	-3.01E-06	1.29E-05	4.93E-02	1.81E-06	6.93E-08	1.47E-02
cD_{04}	-1.13E-06	-6.46E-05	1.48E-02	-1.87E-06	1.27E-05	5.55E-02	1.48E-06	1.43E-04	4.47E-02
cD_{03}	1.26E-05	2.20E-05	6.41E-01	4.29E-06	7.21E-06	2.80E-02	7.07E-06	9.86E-06	2.50E-01
cD_{02}	3.19E-07	-2.58E-05	8.60E-01	3.76E-07	-2.75E-06	2.44E-02	-1.81E-06	-4.26E-06	2.35E+00
cD_{01}	2.97E-06	-1.78E-06	1.36E+00	2.77E-07	-2.28E-05	3.75E-02	-1.88E-07	-1.24E-06	1.31E+00

Table S3. The Mean, Median and Energy values of A2M at each detailed level with three ms-scan configurations using MALDI CSPD LIT-MS.

Detail level	MALDI LIT-MS								
	CENTRAL SECTION								
	RF mode			AC mode			RF + AC mode		
	Mean	Median	Energy	Mean	Median	Energy	Mean	Median	Energy
cD_{11}	9.17E-06	1.40E-05	1.29E-02	-2.63E-04	-3.62E-04	1.08E-01	4.20E-04	5.62E-04	4.57E-01
cD_{10}	-3.07E-05	1.95E-04	4.41E-03	-2.60E-04	1.00E-06	7.08E-02	-7.99E-04	2.89E-04	2.77E-01
cD_{09}	1.14E-04	-3.69E-05	2.38E-03	-1.59E-04	6.53E-05	3.44E-02	-3.71E-04	-4.85E-05	1.50E-01
cD_{08}	8.30E-06	1.37E-05	2.08E-03	-7.87E-05	-7.11E-06	1.64E-02	-1.56E-04	4.84E-05	7.28E-02
cD_{07}	-1.72E-05	-1.44E-05	2.59E-03	-4.81E-05	-1.41E-07	7.24E-03	-9.01E-05	1.76E-05	3.57E-02
cD_{06}	1.42E-05	-5.61E-06	2.37E-03	-1.10E-05	-1.44E-05	5.70E-03	-3.15E-05	2.53E-05	1.10E-02
cD_{05}	6.58E-06	-1.37E-05	9.83E-02	1.73E-05	4.96E-05	1.96E-02	-2.33E-05	1.17E-05	1.36E-02
cD_{04}	1.92E-06	-6.94E-06	3.96E-02	1.79E-06	7.31E-06	2.54E-02	1.11E-05	4.62E-05	1.09E-01
cD_{03}	4.84E-05	2.93E-05	1.65E+00	1.93E-06	2.38E-05	6.82E-02	-1.25E-05	-1.75E-05	1.35E+00
cD_{02}	-4.20E-07	2.70E-05	1.75E+00	-2.96E-06	9.27E-05	7.87E-02	8.19E-06	2.20E-05	6.06E-01
cD_{01}	2.40E-09	2.66E-04	3.50E-01	-1.79E-07	6.14E-04	3.41E-01	6.52E-06	5.20E-04	3.67E-01

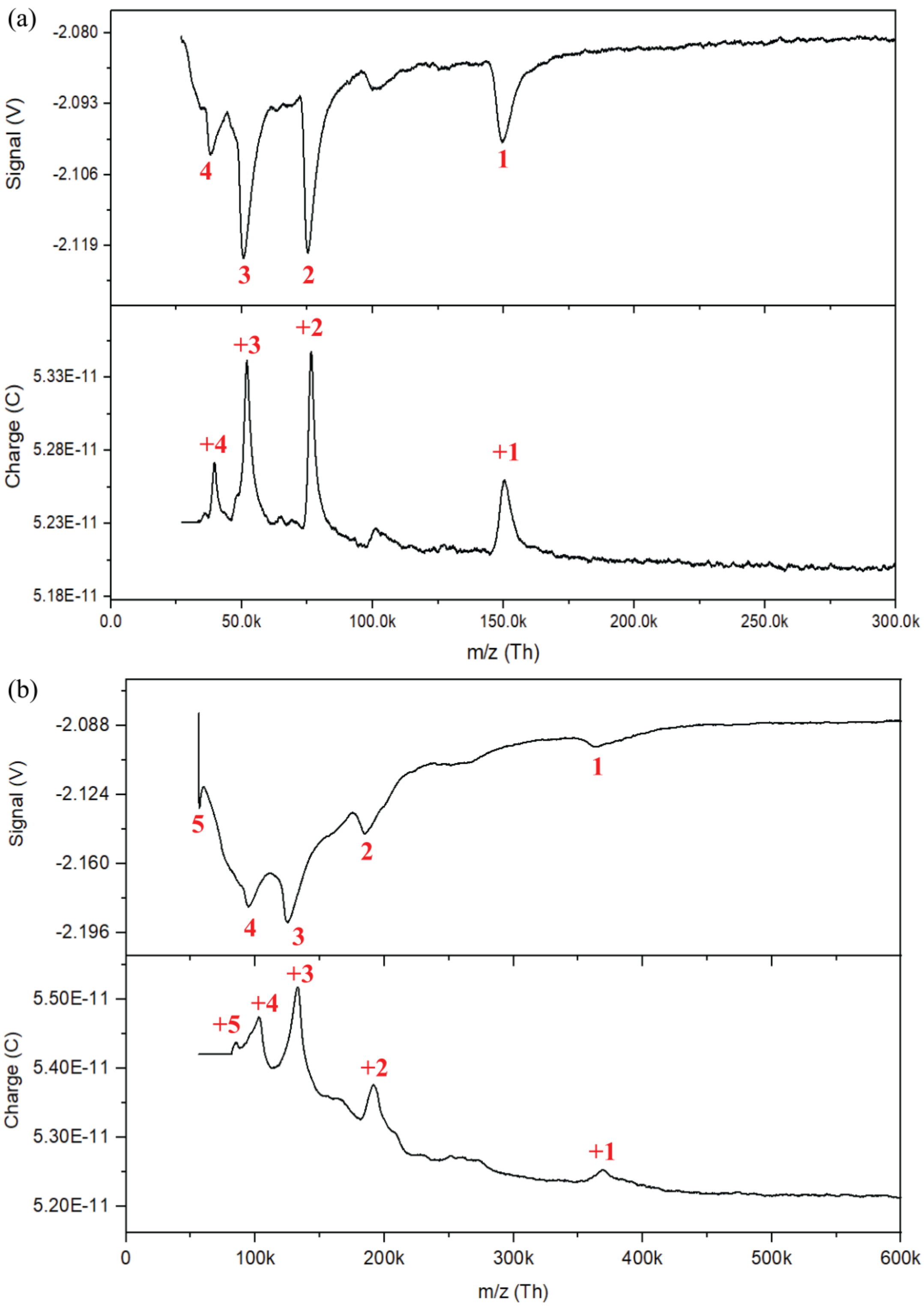

Figure S7. The signal after removal is a strong RF + AC interference, and the mass spectra are converted by CSPD with configuration RF + AC mode. **(a)** IgG sample and **(b)** A2M sample.

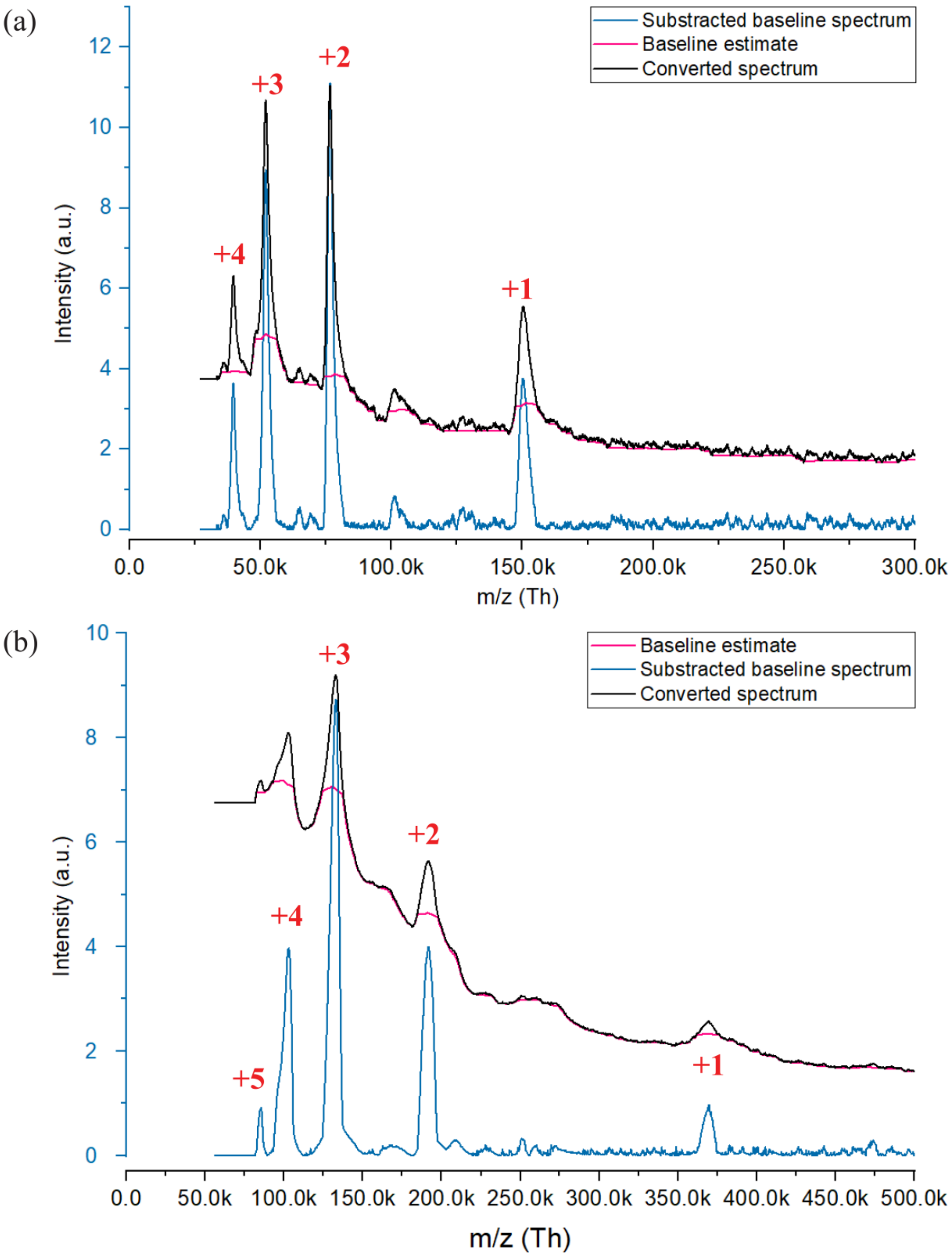

Figure S8. Baseline subtraction in MALDI CSPD LIT-MS with configuration RF + AC mode with Y-unit is arbitrary unit (a.u.). **(a)** IgG sample and **(b)** A2M sample.

Table S4. The attenuation of a total energy at each level corresponds to the threshold setting by the coefficient sigma with the RF mode condition for the IgG sample.

Level	Total of energy in each level after denoising										
	**Energy (σ1)***	**Energy (σ2)**	**Energy (σ3)**	**Energy (σ4)**	**Energy (σ5)**	**Energy (σ6)**	**Energy (σ7)***	**Energy (σ8)**	**Energy (σ9)***	**Energy (σ10)***	**Energy (σ11)**
**cA11**	1.8E+06	1.8E+06	1.8E+06	1.8E+06	1.8E+06	1.8E+06	1.8E+06	1.8E+06	1.8E+06	1.8E+06	1.8E+06
**cD11**	1.3E-02	1.3E-02	1.3E-02	1.3E-02	1.3E-02	1.3E-02	1.3E-02	1.3E-02	1.3E-02	1.3E-02	1.3E-02
**cD10**	4.4E-03	4.4E-03	4.4E-03	4.4E-03	4.4E-03	4.4E-03	4.4E-03	4.4E-03	4.4E-03	4.4E-03	4.4E-03
**cD9**	2.4E-03	2.4E-03	2.4E-03	2.4E-03	2.4E-03	2.4E-03	2.4E-03	2.4E-03	2.4E-03	2.4E-03	2.4E-03
**cD8**	2.1E-03	2.1E-03	2.1E-03	2.1E-03	2.1E-03	2.1E-03	2.1E-03	2.1E-03	2.1E-03	2.1E-03	2.1E-03
**cD7**	0.0E+00	0.0E+00	0.0E+00	4.6E-05	9.8E-05	1.6E-04	0.0E+00	4.6E-05	0.0E+00	0.0E+00	9.8E-05
**cD6**	0.0E+00	0.0E+00	0.0E+00	8.8E-05	1.2E-04	1.2E-04	0.0E+00	8.8E-05	0.0E+00	0.0E+00	1.2E-04
**cD5**	0.0E+00	0.0E+00	0.0E+00	3.8E-05	8.4E-03	7.4E-02	0.0E+00	0.0E+00	0.0E+00	0.0E+00	3.0E-03
**cD4**	0.0E+00	0.0E+00	0.0E+00	0.0E+00	0.0E+00	1.9E-03	0.0E+00	0.0E+00	0.0E+00	0.0E+00	0.0E+00
**cD3**	0.0E+00	2.1E-04	1.3E+00	1.5E+00	1.6E+00	1.6E+00	0.0E+00	1.4E+00	0.0E+00	0.0E+00	1.5E+00
**cD2**	0.0E+00	0.0E+00	2.6E-02	8.0E-01	1.3E+00	1.6E+00	0.0E+00	5.3E-01	0.0E+00	0.0E+00	1.2E+00
**cD1**	0.0E+00	0.0E+00	0.0E+00	3.9E-05	6.5E-03	1.4E-01	0.0E+00	0.0E+00	0.0E+00	0.0E+00	2.5E-03

Table S5. The attenuation of the total energy at each level corresponds to the threshold setting by the coefficient sigma with the AC mode condition for the IgG sample.

**Level**	**Total of energy in each level after denoising**										
	**Energy (σ1)***	**Energy (σ2)***	**Energy (σ3)***	**Energy (σ4)**	**Energy (σ5)**	**Energy (σ6)**	**Energy (σ7)***	**Energy (σ8)***	**Energy (σ9)***	**Energy (σ10)**	**Energy (σ11)**
**cA11**	1.8E+06	1.8E+06	1.8E+06	1.8E+06	1.8E+06	1.8E+06	1.8E+06	1.8E+06	1.8E+06	1.8E+06	1.8E+06
**cD11**	1.8E-02	1.8E-02	1.8E-02	1.8E-02	1.8E-02	1.8E-02	1.8E-02	1.8E-02	1.8E-02	1.8E-02	1.8E-02
**cD10**	2.8E-03	2.8E-03	2.8E-03	2.8E-03	2.8E-03	2.8E-03	2.8E-03	2.8E-03	2.8E-03	2.8E-03	2.8E-03
**cD9**	1.2E-03	1.2E-03	1.2E-03	1.2E-03	1.2E-03	1.2E-03	1.2E-03	1.2E-03	1.2E-03	1.2E-03	1.2E-03
**cD8**	1.2E-03	1.2E-03	1.2E-03	1.2E-03	1.2E-03	1.2E-03	1.2E-03	1.2E-03	1.2E-03	1.2E-03	1.2E-03
**cD7**	0.0E+00	0.0E+00	0.0E+00	0.0E+00	0.0E+00	1.2E-05	0.0E+00	0.0E+00	0.0E+00	1.2E-05	1.1E-04
**cD6**	0.0E+00	0.0E+00	0.0E+00	0.0E+00	0.0E+00	7.2E-06	0.0E+00	0.0E+00	0.0E+00	7.2E-06	2.7E-05
**cD5**	0.0E+00	0.0E+00	0.0E+00	2.8E-04	2.7E-03	3.1E-02	0.0E+00	0.0E+00	5.4E-04	3.3E-02	4.2E-02
**cD4**	0.0E+00	0.0E+00	0.0E+00	2.3E-04	3.2E-03	2.5E-02	0.0E+00	0.0E+00	6.9E-04	2.7E-02	3.7E-02
**cD3**	0.0E+00	0.0E+00	0.0E+00	0.0E+00	0.0E+00	2.2E-04	0.0E+00	0.0E+00	0.0E+00	3.1E-04	2.1E-03
**cD2**	0.0E+00	0.0E+00	0.0E+00	0.0E+00	0.0E+00	2.4E-04	0.0E+00	0.0E+00	0.0E+00	2.9E-04	1.2E-03
**cD1**	0.0E+00	0.0E+00	0.0E+00	0.0E+00	0.0E+00	5.1E-04	0.0E+00	0.0E+00	0.0E+00	6.5E-04	2.6E-03

Table S6. The attenuation of the total energy at each level corresponds to the threshold setting by the coefficient sigma with the RF + AC mode condition for the IgG sample.

**Level**	**TOTAL OF ENERGY IN EACH LEVEL AFTER DENOISING**										
	**Energy (σ1)***	**Energy (σ2)***	**Energy (σ3)**	**Energy (σ4)**	**Energy (σ5)**	**Energy (σ6)**	**Energy (σ7)**	**Energy (σ8)**	**Energy (σ9)**	**Energy (σ10)***	**Energy (σ11)**
**cA11**	1.8E+06	1.8E+06	1.8E+06	1.8E+06	1.8E+06	1.8E+06	1.8E+06	1.8E+06	1.8E+06	1.8E+06	1.8E+06
**cD11**	1.8E-01	1.8E-01	1.8E-01	1.8E-01	1.8E-01	1.8E-01	1.8E-01	1.8E-01	1.8E-01	1.8E-01	1.8E-01
**cD10**	1.4E-02	1.4E-02	1.4E-02	1.4E-02	1.4E-02	1.4E-02	1.4E-02	1.4E-02	1.4E-02	1.4E-02	1.4E-02
**cD9**	1.5E-03	1.5E-03	1.5E-03	1.5E-03	1.5E-03	1.5E-03	1.5E-03	1.5E-03	1.5E-03	1.5E-03	1.5E-03
**cD8**	1.0E-03	1.0E-03	1.0E-03	1.0E-03	1.0E-03	1.0E-03	1.0E-03	1.0E-03	1.0E-03	1.0E-03	1.0E-03
**cD7**	0.0E+00	0.0E+00	0.0E+00	0.0E+00	0.0E+00	4.4E-05	0.0E+00	0.0E+00	0.0E+00	0.0E+00	0.0E+00
**cD6**	0.0E+00	0.0E+00	0.0E+00	0.0E+00	0.0E+00	1.5E-05	0.0E+00	0.0E+00	0.0E+00	0.0E+00	0.0E+00
**cD5**	0.0E+00	0.0E+00	0.0E+00	0.0E+00	0.0E+00	1.3E-03	0.0E+00	0.0E+00	0.0E+00	0.0E+00	0.0E+00
**cD4**	0.0E+00	0.0E+00	0.0E+00	5.0E-05	8.5E-04	2.1E-02	0.0E+00	0.0E+00	0.0E+00	0.0E+00	0.0E+00
**cD3**	0.0E+00	0.0E+00	6.1E-04	9.7E-02	1.6E-01	2.2E-01	0.0E+00	0.0E+00	0.0E+00	0.0E+00	0.0E+00
**cD2**	0.0E+00	0.0E+00	1.3E+00	2.1E+00	2.2E+00	2.3E+00	8.5E-01	4.1E-01	4.0E-04	0.0E+00	7.3E-01
**cD1**	0.0E+00	0.0E+00	4.4E-01	9.2E-01	1.0E+00	1.2E+00	2.2E-01	7.6E-02	1.0E-03	0.0E+00	1.8E-01

Table S7. The attenuation of the total energy at each level corresponds to the threshold setting by the coefficient sigma with RF mode condition for the A2M sample.

**Level**	**Total of energy in each level after denoising**										
	**Energy (σ1)***	**Energy (σ2)**	**Energy (σ3)**	**Energy (σ4)**	**Energy (σ5)**	**Energy (σ6)**	**Energy (σ7)**	**Energy (σ8)**	**Energy (σ9)***	**Energy (σ10)***	**Energy (σ11)***
**cA11**	9.7E+05	9.7E+05	9.7E+05	9.7E+05	9.7E+05	9.7E+05	9.7E+05	9.7E+05	9.7E+05	9.7E+05	9.7E+05
**cD11**	1.5E-02	1.5E-02	1.5E-02	1.5E-02	1.5E-02	1.5E-02	1.5E-02	1.5E-02	1.5E-02	1.5E-02	1.5E-02
**cD10**	1.5E-03	1.5E-03	1.5E-03	1.5E-03	1.5E-03	1.5E-03	1.5E-03	1.5E-03	1.5E-03	1.5E-03	1.5E-03
**cD9**	1.1E-03	1.1E-03	1.1E-03	1.1E-03	1.1E-03	1.1E-03	1.1E-03	1.1E-03	1.1E-03	1.1E-03	1.1E-03
**cD8**	4.9E-04	4.9E-04	4.9E-04	4.9E-04	4.9E-04	4.9E-04	4.9E-04	4.9E-04	4.9E-04	4.9E-04	4.9E-04
**cD7**	0.0E+00	0.0E+00	0.0E+00	0.0E+00	1.7E-05	0.0E+00	0.0E+00	0.0E+00	0.0E+00	0.0E+00	0.0E+00
**cD6**	0.0E+00	0.0E+00	0.0E+00	0.0E+00	0.0E+00	0.0E+00	0.0E+00	0.0E+00	0.0E+00	0.0E+00	0.0E+00
**cD5**	0.0E+00	0.0E+00	0.0E+00	0.0E+00	0.0E+00	0.0E+00	0.0E+00	0.0E+00	0.0E+00	0.0E+00	0.0E+00
**cD4**	0.0E+00	0.0E+00	0.0E+00	2.1E-04	1.9E-03	2.0E-04	9.4E-04	1.3E-04	0.0E+00	0.0E+00	0.0E+00
**cD3**	0.0E+00	3.0E-02	4.5E-01	5.9E-01	6.2E-01	5.9E-01	6.1E-01	5.7E-01	0.0E+00	0.0E+00	0.0E+00
**cD2**	0.0E+00	1.6E-03	3.7E-01	7.3E-01	8.0E-01	7.3E-01	7.8E-01	6.6E-01	0.0E+00	0.0E+00	0.0E+00
**cD1**	0.0E+00	3.4E-02	4.2E-01	1.1E+00	1.2E+00	1.1E+00	1.2E+00	1.0E+00	0.0E+00	0.0E+00	0.0E+00

Table S8. The attenuation of the total energy at each level corresponds to the threshold setting by the coefficient sigma with AC mode condition for the A2M sample.

**Level**	**Total of energy in each level after denoising**										
	**Energy (σ1)***	**Energy (σ2)***	**Energy (σ3)**	**Energy (σ4)**	**Energy (σ5)**	**Energy (σ6)**	**Energy (σ7)**	**Energy (σ8)**	**Energy (σ9)**	**Energy (σ10)***	**Energy (σ11)**
**cA11**	1.8E+06	1.8E+06	1.8E+06	1.8E+06	1.8E+06	1.8E+06	1.8E+06	1.8E+06	1.8E+06	1.8E+06	1.8E+06
**cD11**	1.8E-01	1.8E-01	1.8E-01	1.8E-01	1.8E-01	1.8E-01	1.8E-01	1.8E-01	1.8E-01	1.8E-01	1.8E-01
**cD10**	1.4E-02	1.4E-02	1.4E-02	1.4E-02	1.4E-02	1.4E-02	1.4E-02	1.4E-02	1.4E-02	1.4E-02	1.4E-02
**cD9**	1.5E-03	1.5E-03	1.5E-03	1.5E-03	1.5E-03	1.5E-03	1.5E-03	1.5E-03	1.5E-03	1.5E-03	1.5E-03
**cD8**	1.0E-03	1.0E-03	1.0E-03	1.0E-03	1.0E-03	1.0E-03	1.0E-03	1.0E-03	1.0E-03	1.0E-03	1.0E-03
**cD7**	0.0E+00	0.0E+00	0.0E+00	0.0E+00	0.0E+00	4.4E-05	0.0E+00	0.0E+00	0.0E+00	0.0E+00	0.0E+00
**cD6**	0.0E+00	0.0E+00	0.0E+00	0.0E+00	0.0E+00	1.5E-05	0.0E+00	0.0E+00	0.0E+00	0.0E+00	0.0E+00
**cD5**	0.0E+00	0.0E+00	0.0E+00	0.0E+00	0.0E+00	1.3E-03	0.0E+00	0.0E+00	0.0E+00	0.0E+00	0.0E+00
**cD4**	0.0E+00	0.0E+00	0.0E+00	5.0E-05	8.5E-04	2.1E-02	0.0E+00	0.0E+00	0.0E+00	0.0E+00	0.0E+00
**cD3**	0.0E+00	0.0E+00	6.1E-04	9.7E-02	1.6E-01	2.2E-01	0.0E+00	0.0E+00	0.0E+00	0.0E+00	0.0E+00
**cD2**	0.0E+00	0.0E+00	1.3E+00	2.1E+00	2.2E+00	2.3E+00	8.5E-01	4.1E-01	4.0E-04	0.0E+00	7.3E-01
**cD1**	0.0E+00	0.0E+00	4.4E-01	9.2E-01	1.0E+00	1.2E+00	2.2E-01	7.6E-02	1.0E-03	0.0E+00	1.8E-01

Table S9. The attenuation of the total energy at each level corresponds to the threshold setting by the coefficient sigma with the RF + AC mode condition for the A2M sample.

Level	Total of energy in each level after denoising										
	**Energy (σ1)***	**Energy (σ2)**	**Energy (σ3)**	**Energy (σ4)**	**Energy (σ5)**	**Energy (σ6)**	**Energy (σ7)**	**Energy (σ8)**	**Energy (σ9)***	**Energy (σ10)**	**Energy (σ11)**
**cA11**	9.8E+05	9.8E+05	9.8E+05	9.8E+05	9.8E+05	9.8E+05	9.8E+05	9.8E+05	9.8E+05	9.8E+05	9.8E+05
**cD11**	4.6E-01	4.6E-01	4.6E-01	4.6E-01	4.6E-01	4.6E-01	4.6E-01	4.6E-01	4.6E-01	4.6E-01	4.6E-01
**cD10**	2.8E-01	2.8E-01	2.8E-01	2.8E-01	2.8E-01	2.8E-01	2.8E-01	2.8E-01	2.8E-01	2.8E-01	2.8E-01
**cD9**	1.5E-01	1.5E-01	1.5E-01	1.5E-01	1.5E-01	1.5E-01	1.5E-01	1.5E-01	1.5E-01	1.5E-01	1.5E-01
**cD8**	7.3E-02	7.3E-02	7.3E-02	7.3E-02	7.3E-02	7.3E-02	7.3E-02	7.3E-02	7.3E-02	7.3E-02	7.3E-02
**cD7**	3.3E-02	3.4E-02	3.5E-02	3.5E-02	3.5E-02	3.5E-02	3.5E-02	3.5E-02	3.2E-02	3.4E-02	3.5E-02
**cD6**	6.2E-03	6.2E-03	7.0E-03	7.1E-03	7.1E-03	7.1E-03	7.1E-03	6.7E-03	6.2E-03	6.2E-03	7.0E-03
**cD5**	0.0E+00	6.3E-04	8.2E-04	9.1E-04	2.0E-03	9.1E-04	9.1E-04	8.2E-04	0.0E+00	6.3E-04	8.2E-04
**cD4**	3.2E-03	3.2E-03	2.5E-02	6.0E-02	7.8E-02	5.4E-02	4.8E-02	4.5E-03	3.2E-03	3.2E-03	1.2E-02
**cD3**	1.1E-03	1.1E-03	1.0E+00	1.3E+00	1.3E+00	1.2E+00	1.2E+00	4.8E-01	0.0E+00	1.1E-03	8.3E-01
**cD2**	0.0E+00	8.4E-04	4.0E-02	3.0E-01	4.6E-01	2.6E-01	2.0E-01	1.3E-03	0.0E+00	8.4E-04	7.7E-03
**cD1**	0.0E+00	6.0E-04	6.6E-04	2.1E-02	1.1E-01	1.1E-02	5.6E-03	6.0E-04	0.0E+00	6.0E-04	6.0E-04

Table S10. The S/N values of IgG and A2M sample in RF mode, AC mode and RF+AC mode.

Sample	Mode	Raw Signal	Signal Without Noise	Difference
**IgG**	RF	10.26	43.77	33.51
	AC	13.76	39.46	25.69
	RF + AC	16.98	70	53.02
**A2M**	RF	11.8	93.06	81.26
	AC	12.03	65	52.97
	RF + AC	19.03	246.33	227.31

REFERENCES

1. Snyder, D. T.; Pulliam, C. J.; Cooks, R. G., Linear mass scans in quadrupole ion traps using the inverse Mathieu q scan. *Rapid Communications in Mass Spectrometry* **2016,** *30* (22), 2369-2378.
2. Chou, S.-W.; Lee, Y.-K.; Hsiao, Y.-T.; Fan, L.-C.; Cheng, C.-Y.; Lee, P.-D.; Tseng, Y.-H., *Charge-sensing particle detector (CSPD): a sensitivity-enhanced Faraday cup*. 2019.
3. Brigham, E. O.; Morrow, R. E., The fast Fourier transform. *IEEE Spectrum* **1967,** *4* (12), 63-70.
4. Zhongde, W., Fast algorithms for the discrete W transform and for the discrete Fourier transform. *IEEE Transactions on Acoustics, Speech, and Signal Processing* **1984,** *32* (4), 803-816.
5. Stéphane, M., CHAPTER 8 - Wavelet Packet and Local Cosine Bases. In *A Wavelet Tour of Signal Processing (Third Edition)*, Stéphane, M., Ed. Academic Press: Boston, 2009; pp 377-434.
6. Stéphane, M., CHAPTER 7 - Wavelet Bases. In *A Wavelet Tour of Signal Processing (Third Edition)*, Stéphane, M., Ed. Academic Press: Boston, 2009; pp 263-376.
7. Daubechies, I., *Ten Lectures on Wavelets*. Society for Industrial and Applied Mathematics: 1992.
8. Zuo, L. Q.; Sun, H. M.; Mao, Q. C.; Liu, X. Y.; Jia, R. S., Noise Suppression Method of Microseismic Signal Based on Complementary Ensemble Empirical Mode Decomposition and Wavelet Packet Threshold. *IEEE Access* **2019,** *7*, 176504-176513.
9. Jang, Y. I.; Sim, J. Y.; Yang, J.-R.; Kwon, N. K. The Optimal Selection of Mother Wavelet Function and Decomposition Level for Denoising of DCG Signal *Sensors* [Online], 2021.
10. Gao, R. X.; Yan, R. In *Wavelets: Theory and Applications for Manufacturing*, 2010.
11. Entropy, Relative Entropy, and Mutual Information. In *Elements of Information Theory*, 2005; pp 13-55.
12. Yang, Q.; Wang, J. Multi-Level Wavelet Shannon Entropy-Based Method for Single-Sensor Fault Location *Entropy* [Online], 2015, p. 7101-7117.
13. Réfrégier, P., Information and Fluctuations. In *Noise Theory and Application to Physics: From Fluctuations to Information*, Réfrégier, P., Ed. Springer New York: New York, NY, 2004; pp 109-136.
14. Eilers, P.; Boelens, H., Baseline Correction with Asymmetric Least Squares Smoothing. *Unpubl. Manuscr* **2005**.
15. Zhang, Z.-M.; Chen, S.; Liang, Y.-Z., Baseline correction using adaptive iteratively reweighted penalized least squares. *Analyst* **2010,** *135* (5), 1138-1146.
16. Stanford, T. E.; Bagley, C. J.; Solomon, P. J., Informed baseline subtraction of proteomic mass spectrometry data aided by a novel sliding window algorithm. *Proteome Science* **2016,** *14* (1), 19.
